# Enhancement of solar evacuated tube unit filled with nanofluid implementing three lobed storage unit equipped with fins

**DOI:** 10.1038/s41598-024-58276-4

**Published:** 2024-04-04

**Authors:** S. M. Mousavi, M. Sheikholeslami

**Affiliations:** 1 Department of Mechanical Engineering, Babol Noshirvani University of Technology, Babol, Islamic Republic of Iran; 2 Renewable Energy Systems and Nanofluid Applications in Heat Transfer Laboratory, Babol Noshirvani University of Technology, Babol, Islamic Republic of Iran

**Keywords:** NEPCM, Storage tank, Nanofluid, Evacuated tube, Thermal energy, Melting, Energy storage, Nanoscience and technology, Nanoscale materials

## Abstract

This study discusses an evacuated tube collector-type solar water heater (ETCSWH) using a phase change material (PCM) chamber with fins, nanofluid, and nano-enhanced phase change material (NEPCM). First, the charging phenomena in a horizontal triplex tube heat exchanger (TTHX) equipped with fins, natural convection, and an ETCSWH system without PCM is simulated to validate the solution. The impact of adding fins and nanoparticles with a volume fraction of 3% of Al_2_O_3_ and Cu to paraffin wax and water-based fluid, respectively, on the unit's efficiency has been examined. The proposed system for the PCM melting process, heat storage, fluid flow behavior in the system, and velocity distribution and temperature contour in the storage tank and three parts of the absorber tube have been evaluated using ANSYS FLUENT software in a three-dimensional and transient simulation. The results show that Case 8 has improved by 39.7% compared to Case 1 and Case 4 by 5.2% compared to Case 1 within 4 h of the melting process. Also, Case 8 with a 43% and 6.4% shorter melting time than Cases 1 and 5 has the best performance and the greatest heat transfer rate. The productivity of the ETCSWH system is considerably enhanced by the use of fins, NEPCM, and nanofluid.

## Introduction

The population of the planet is growing rapidly every year, and factors such as industrial development, fast urbanization, and others contribute to this growth. The environmental pollution, the non-renewability of fossil fuels, and the negative impacts of their use, such as the rising greenhouse gas emissions, make it essential to address this global issue in the present day. This requires using new methods to produce clean and renewable fuels, such as solar energy, water, wave, wind, nuclear, and thermal. Solar energy is one of these abundant and eco-friendly sources of power^1,2^. The nations face a difficult challenge in meeting the growing energy demand without further harming the environment^3^. Solar energy is a great option that helps reduce the reliance on fossil energy. Domestic solar water heating systems (DSWHS) are one of the most common uses of solar energy. However, there are some issues with its use. The biggest one is ensuring that there is enough hot water available throughout the day. The efficiency of solar water heating systems also needs to be improved, which requires thermal energy storage (TES) technology^4^. Solar water collectors are an example of a solar energy application that uses solar energy to heat water to a suitable temperature for both domestic and industrial use. One of the most popular types of solar water collectors is the evacuated tube model, which has gained popularity recently due to its tubular structure, which follows the sun throughout the day and absorbs more sunlight than flat plate collectors (FPC)^5^. Solar water heating (SWH) systems aim to heat water and produce steam. This reduces the emissions of greenhouse gases. SWH systems are essential applications for solar energy^6,7^. One of the most popular kinds of solar collectors is the evacuated tube collector (ETC), especially the glass ETSC. ETCs can collect solar energy in various ways. There are three different types of solar collectors: concentrating collectors, FPCs, and ETCs. An ETSC consists of a series of borosilicate glass tubes connected to a manifold. Each glass tube is surrounded by a second glass tube, and there is an annular space between them to reduce heat loss. The working fluid in ETSC is a fluid that absorbs heat from solar radiation. Recently, some studies have used nanofluids to evaluate and improve the ETSC's performance. Nanofluids are formed by mixing nanoparticles smaller than 100 nm into a base fluid^8,9^. ETSCs can be divided into 3 categories based on heat extraction methods: U-type, heat pipe ETCs, and thermosyphon. Researchers have focused on (water-in-glass) WIG or thermosyphon models because of their easy maintenance, simplicity, and suitable thermal efficiency. The thermosyphon system uses the property that heated water rises (a decrease in density). In this system, a storage tank is placed above the collector. Water flows naturally to the tank because it becomes lighter when heated. A natural convection occurs in the system as colder water moves to the lowest point of the collector^10,11^. Materials that undergo phase changes can store and use latent energy. These materials can retain heat near their melting point by changing phases after absorbing heat from a heat source like the sun. These materials can be used as thermal absorbers and thermal storage tanks in construction, civil engineering, and other fields. The energy that these materials can hold in themselves is five to fourteen times more than the energy that can be used by materials like stone or water. According to studies^12^, the productivity is improved by the use of PCMs. PCM can release thermal energy to the environment after absorbing it at high temperatures. The PCM's temperature rises until it reaches its melting point. The PCM then converts the heat from a solid to a liquid phase while keeping a constant temperature. The PCM temperature will then rise by increasing its temperature after all the materials have melted^13^. PCM has poor thermal performance due to its low heat conductivity. To improve PCM's thermal performance, nanoparticles with high thermal conductivity, such as metallic or non-metallic ones, can be mixed. PCMs that are modified with nanoparticles are called NEPCMs. Nanoparticles can change the thermophysical features and augment the productivity^14^. The cost, operating conditions, and thermal performance of ETSCs are much better than those of FPCs at temperatures above 60 °C^15^. Many numerical and experimental studies have examined the heat handling of ETSCs. Collector efficiency^11,16,17^ is affected by various factors, such as collector inclination, solar radiation, flow rate, HTF, longitude and latitude, use of fins, use of nanoparticles, and geometrical parameters. Sharafeldin and Grof^18^ showed the importance and effectiveness of nanofluid in ETC. The reports with 0.03 nanoparticle mass fraction and flow rate of 0.8 L per minute increased the T_out_ by 50%. Kumar and Mylsamy^19^ investigated an advanced type of NEPCM by dispersing CeO_2_ nanoparticles in paraffin at three fractions of 0.5, 1.0 and 2.0. The outputs indicated that the addition of CeO_2_ nano-powders significantly increased the thermal storage capabilities. Manirathnam et al.^20^ investigated the energy storage capacity of a solar water heater using PCM and NECPCM as nanocomposite. Performance results in thermosyphon flow after one day showed that the efficiency of the system for three Cases was 33.8, 38.3 and 41.7%, respectively, and the efficiency for no PCM, PCM and NCPCM was 1.78, 2.18 and 3.23%, respectively. Uniyal et al.^21^ scrutinized the melting and heat transfer features of three PCMs in an ETC collector during the day, Lauric acid melted the fastest, followed by paraffin wax and stearic acid.

Rinawa et al.^22^ prepared twin/paraffin nanoparticles by adding 1 mass% of the equivalent composition of SiO_2_ and CuO particles to paraffin wax to increase the energy storage of ETCSWH. The research showed that increasing the water temperature with twin nano paraffins and more paraffin improved the SWH collector's thermal storage capacity. The findings revealed that the temperatures of paraffin and water increased by 8.8 and 11.7 degrees Celsius, respectively, due to nano and paraffin. Núñez et al.^23^ compared the thermodynamic performance and entropy of a WIG-ETSC model using computational fluid dynamics and water as the circulating fluid. The results showed that the application of nanofluid improved the temperature and output speed of the vacuum tube solar collector. Also, using nanofluids improved thermal and energy efficiency. Deshmukh et al.^24^ investigated the convective heat exchange capabilities of a U-tube ETSC with tin nanofluid. They dissolved various volume concentrations of tin nanoparticles with a size range of 40 to 50 nm in distilled water. The outcomes showed that the solar collector yield of the ETC reached 70.9%. Nawsud et al.^25^ analyzed the efficiency of combining nanoparticles with PCM more carefully. They proved that using NEPCM and nanofluid increased the energy efficiency and exergy of the intended collector. Moreover, by using various fins and extended surfaces suitable for the increased surface area and higher thermal conductivity coefficient, the heat transfers between the PCM, the HTF, and the heat source can be enhanced^26^. Pawar et al.^27^ compared a Heat pipe unit with PCM and porous copper metal in the discharge pipe with a standard HPETC system. The outputs demonstrated that the PCM and copper metal enhanced the peak temperature by about 21 °C at the highest solar radiation. They also maintained a higher fin temperature of 36.1 °C and a higher T_out_ of 8–11 °C after sunset than the standard system. Khedher et al.^28^ performed a simulation to analyze a LHTES geometry model in a vertical cylindrical state using NEPCM with internal and external fins, including nanoparticles, 1% Carbon nanotube (CNT), and 2% Al_2_O_3_. The use of NEPCM with 1 weight percent of CNT nanoparticles dispersed in finned PCM decreased the discharge time by 89% and the charging time by 84%. Bouadila et al.^29^ analyzed various modes of ETSC. They proved that the ETSC collector was improved by using reflectors, fins, phase change materials, nanofluids and adding nanoparticles.

The main goal of current investigation is to analyze an ETCSWH operating under thermosyphon flow, featuring an energy storage tank equipped with tree-type copper fins and NEPCM containing a 3% volume dispersion of Al_2_O_3_ nanoparticles in paraffin wax (RT30). The system, inclined at a 45-degree angle, explores the distribution of Cu nanoparticles with a 3% volume fraction in pure water as the base fluid. The proposed setup not only explores the impact of various modes on the melting characteristics of integrated PCMs in the ETCSWH system's storage tank but also delves into energy storage, fluid flow behavior, velocity distribution, and temperature contours in the storage tank and three distinct sections of the absorber tube. This evaluation is conducted for different time conditions, comparing pure water and copper/water nanofluids through a 3D simulation utilizing the FVM. The analysis specifically focuses on transient and laminar flow over a 4-h period. Despite the existing literature on solar water heating systems, there is a research gap in comprehensively exploring the interplay of different modes on the melting characteristics of integrated PCMs within the storage tank of ETCSWH systems. Additionally, limited studies have investigated the combined effects of tree-type copper fins and NEPCM dispersion with Al_2_O_3_ nanoparticles. This study aims to address these gaps and contribute valuable insights to the field. This study introduces a novel approach by incorporating tree-type copper fins and NEPCM with Al_2_O_3_ nanoparticles into an ETCSWH system. The detailed examination of various modes on PCM melting characteristics, coupled with the assessment of energy storage, fluid flow behavior, and temperature distribution, adds a unique dimension to the existing body of research. The exploration of copper/water nanofluids further enhances the novelty of this work, providing a comprehensive analysis for improved understanding and potential advancements in solar water heating technology.

## Problem description

**Figure 1:**
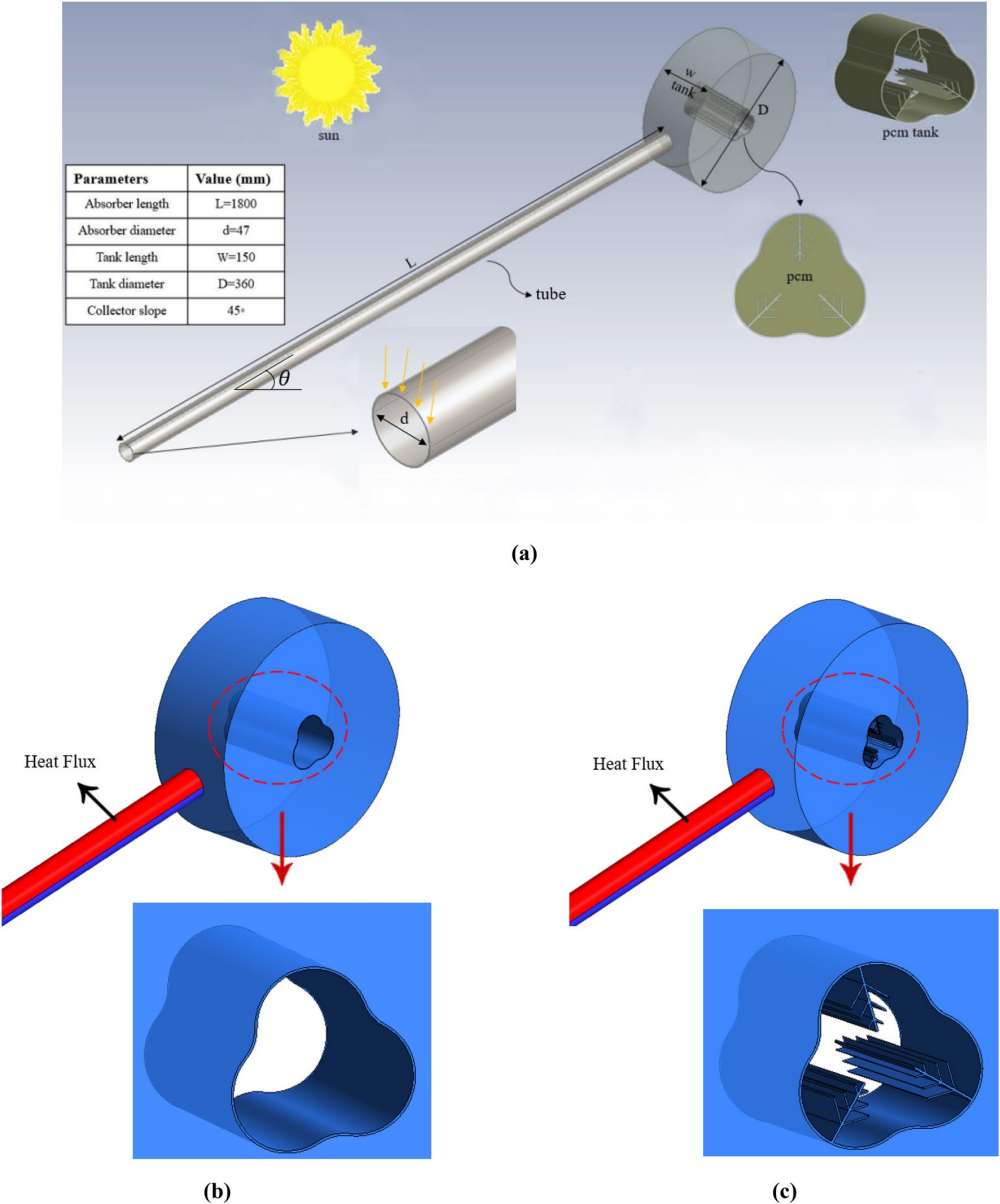
(**a**) The suggested geometry of the system with an energy storage tank. (**b**) Without fins. (**c**) With fins.

Figure 1 shows a three-dimensional geometry of an ETCSWH with one open end and one closed end, along with a table of its geometric dimensions. Figure 1a,b show two geometries with and without fins for this study. The proposed system is simulated with the FVM using the Boussinesq approximation that works under thermosyphon flow, which has the most optimal inclination angle of 45 degrees^30^. The thermosiphon system uses the property of water rising when the absorber tube absorbs the sun's heat and heats up (decrease in density). In this study, a storage tank is placed above the collector. The water inside the absorber tube becomes lighter as it heats up by absorbing solar radiation, and its density decreases. It naturally flows to the highest point inside the storage tank. Colder water, because it is denser, flows to the lowest point of the collector, and a natural flow occurs in the ETCSWH system. The circulation in the system stops when the temperature of the collector fluid is lower than the temperature inside the storage tank. Otherwise, the water flow continues in the collector.

Encapsulated paraffin wax (RT30) is located in a special chamber integrated into the tank that holds heated liquids in the middle of the tank as a thermal storage medium. For better heat transfer and faster melting process, tree fins are used in the PCM chamber. Aluminium oxide (Al_2_O_3_) and copper (Cu) are chosen as nanoparticle materials with a fraction of 0.03 to increase heat transfer for PCM and working fluid (water), respectively. Table 1 displays the properties of water, nanoparticles, and PCM.

**Table 1:** Material properties^10,11,31,32^.

Material	RT30		Water	Cu	Al_2_O_3_	Units
$\rho$	$\rho_{s}$	870	998.2	6320	3970	(kg/m^3^)
$-$	$\rho_{l}$	760				
$C_{P}$	$C_{{P_{S} }}$	2400	4182	531.8	765	(J/(kg K))
	$C_{{P_{l} }}$	1800				
$K$	0.2		0.6	76.5	40	(W/(m K)
$\beta$	0.0006		0.00333	0.0000180	0.0000085	(1/K)
$\mu$	0.0038		0.001003			(kg/m s)
$T_{s}$	300.15					K
$T_{l}$	301.15					K
$L$	222,000					(J/kg)

## Numerical simulation

In this research, a 3D model of ETCSWH is simulated using the FVM method in ANSYS-FLUENT software. Figure 1 also shows the dimensions of the examined geometry. We know that the maximum amount of sunlight during the day is from 11 am to 3 pm. Therefore, it has been simulated with the average maximum solar radiation of 900 watts per square meter for a period of 4 h.

### Calculation method and validation

**Figure 2:**
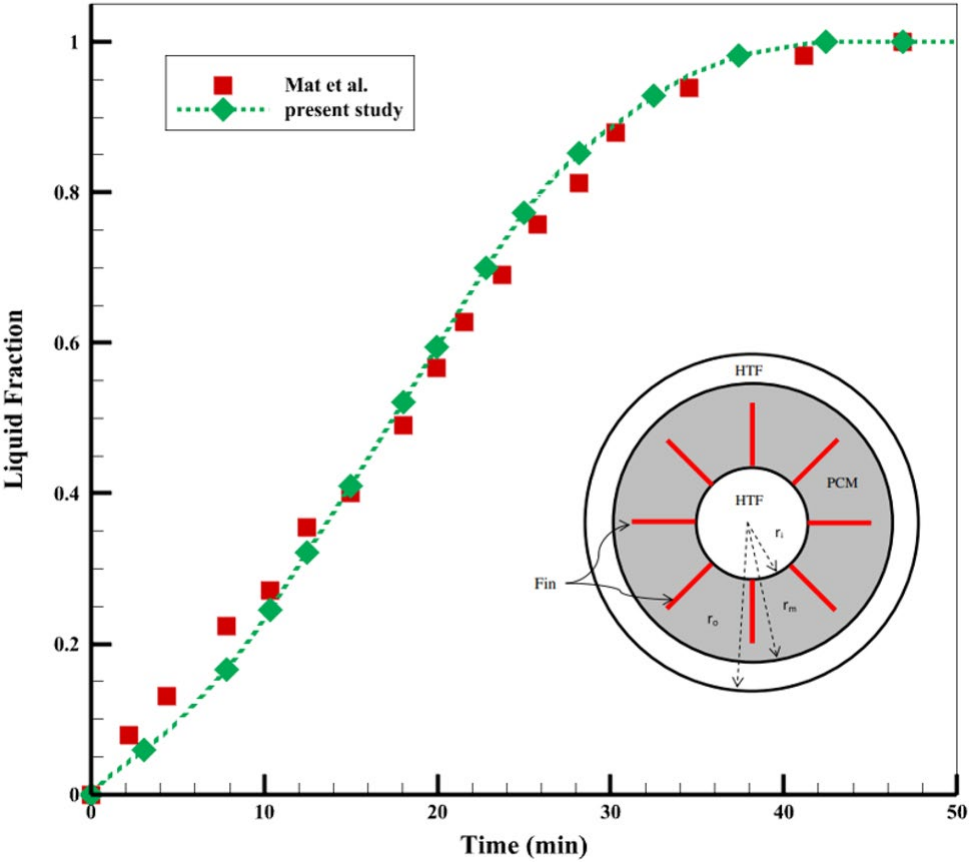
The validation of the PCM melting process^33^.

The velocity and pressure coupled algorithm solves the governing equations in this research. The pressure interpolation uses PRESTO and the gradient uses Green-Gauss node-based. The 2nd order upwind scheme discretizes the momentum term. The Boussinesq approximation computes the density difference in the absorber tube's slope. The testing fluid is incompressible, laminar, and steady. The simulation process is transient and time-dependent, as the previous section mentioned, and the modeling time is 4 h. Energy storage reduces temperature fluctuations, eliminates the energy production and consumption gap, and enhances solar collector performance. The middle tube in a three-tube converter with internal fins validates PCM melting. Figure 2 illustrates that the outputs are close to the PCM melting process^33^.

**Figure 3:**
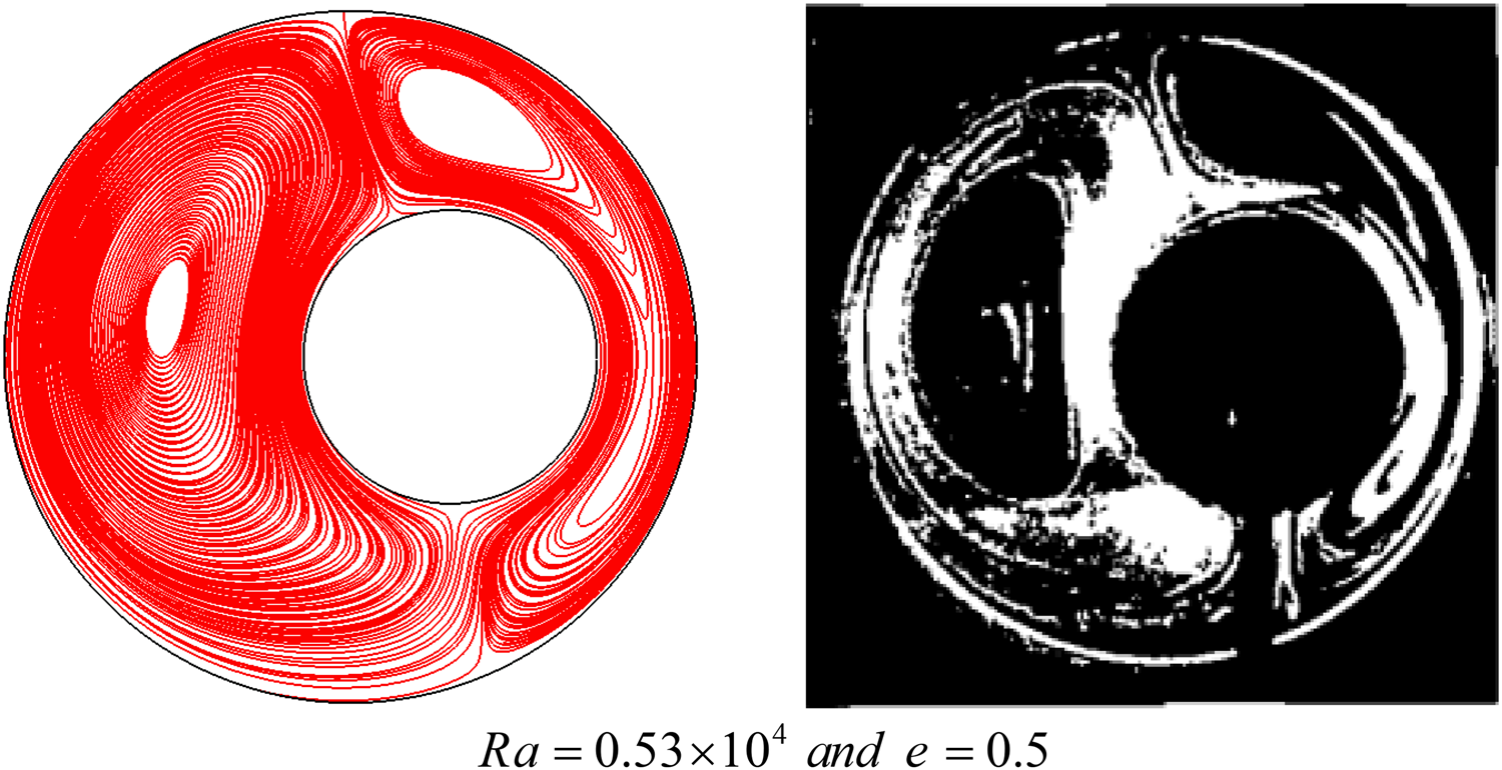
The evaluation of the correctness of the simulation of the this work by comparing the left image with the experimental and numerical outputs in the right image^34,35^.

Figure 3 examines a cavity in the annular region to see the effects of natural convection. Numerical flow lines have been compared with experimental work at $Ra = 0.53 \times 10^{4}$ and eccentricity $e = 0.5$ and $pr = 0.7$. The reported results show that the simulated flow lines are in good agreement with the experimental outcomes^34,35^.

**Figure 4:**
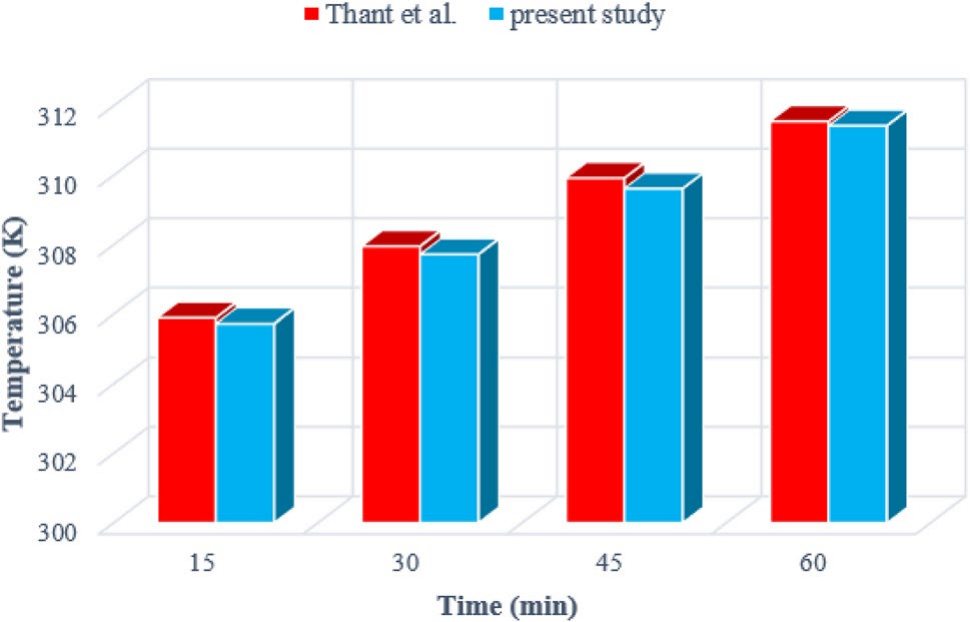
The comparison of the current outputs with the data obtained by Thant et al.^34^.

Validation of a numerical study is important because it can verify the accuracy of the work done. To validate the current study, the results obtained from the present simulation at 15, 30, 45, and 60 min were compared with the outputs reported by Thant et al.^36^. Figure 4 shows that the data obtained for the average tank temperature during one hour of simulation match the work done with very little error. According to the mentioned information, the validation of the current study is confirmed.

### Mathematical modeling

Solving the equations together for an incompressible fluid with a temperature-dependent density is the challenge in this fluid-thermal study. The Boussinesq model is utilized for buoyant flows where the density variation is only affected by small temperature differences. Boussinesq approximation was selected for natural convection in ETC tubes in CFD simulations, and convection characteristics, except for density, were assumed to be constant in this method. The mathematical model are as follows^37^.

1 $$\frac{{\partial \rho_{nf} }}{\partial t} + \nabla \cdot (\rho_{nf} \mathop u\limits^{ \to } ) = 0$$

2 $$\frac{{\rho_{nf} \partial (\mathop u\limits^{ \to } )}}{\partial t} + \rho_{nf} \mathop u\limits^{ \to } (\mathop u\limits^{ \to } \cdot \nabla ) = \nabla \cdot [ - \mathop p\limits^{ \to } + \mu_{nf} (\nabla \mathop u\limits^{ \to } )] + \mathop F\limits^{ \to }$$

3 $$\rho_{nf} C_{{P_{nf} }} \frac{\partial T}{{\partial t}} + \rho_{nf} C_{{P_{nf} }} \mathop u\limits^{ \to } \cdot \nabla T = k_{nf} \nabla^{2} T$$

In the equation above, $\mathop F\limits^{ \to }$ is buoyancy force whose formula the is given below^38^:

4 $$\mathop F\limits^{ \to } = - g\rho_{0} (1 - \beta_{nf} \nabla T)$$

The Boussinesq approximation is defined by Eq. (5) ^38^:

5 $$\rho = \rho_{0} \left[ {1 - \beta_{nf} \left( {T - T_{0} } \right)} \right] = \rho_{0} (1 - \beta_{nf} \nabla T)$$

where $\beta_{nf}$ is the volume expansion coefficient and $\rho_{0}$ is the fluid density at temperature $T_{0}$.

The below equations were utilized to simulate PCM^33^:

6 $$\frac{\partial }{\partial t}\rho_{nepcm} + \nabla \cdot (\rho_{nepcm} \mathop u\limits^{ \to } ) = 0$$

7 $$\frac{\partial }{\partial t}(\rho_{nepcm} \mathop u\limits^{ \to } ) + \nabla \cdot (\rho_{nepcm} \mathop u\limits^{ \to } \mathop u\limits^{ \to } ) = (\mu_{nepcm} \nabla^{2} \mathop u\limits^{ \to } - \nabla p + \rho_{nepcm} \mathop g\limits^{ \to } ) + \mathop S\limits^{ \to }$$

8 $$\frac{\partial }{\partial t}(\rho_{nepcm} h) + \nabla \cdot (\rho_{nepcm} \mathop u\limits^{ \to } h) = \nabla .(k_{nepcm} \nabla T)$$

In this equation, $\rho$ stands for PCM (RT30) density, *p *for pressure, $\mathop u\limits^{ \to }$ for velocity, $\mu$ for dynamic viscosity, *g *for acceleration due to gravity, and *h* for sensible enthalpy.

Sensible enthalpy can be written as follows^33^:

9 $$h = \int_{{T_{ref} }}^{T} {C_{{p_{nepcm} }} } dT + h_{ref}$$

Enthalpy *H *is defined by the following equation^33^:

10 $$H = \Delta H + h$$

where $h_{ref}$ is the reference enthalpy at temperature $T_{ref}$. The latent heat of the PCM is represented by $\Delta H,$ the latent heat quantity, which varies from zero (for a solid) to L (for a liquid). When the temperature is $T_{s} < T < T_{l}$ and $\gamma$ is the fraction that melts when a substance changes from a solid to a liquid, and it is given by^33^:

11 $$\Delta H = \gamma L$$

12 $$\begin{array}{*{20}l} {\gamma = 0} \hfill & {if\;T_{s} > T} \hfill \\ {\gamma = 1} \hfill & {if\;T_{l} < T} \hfill \\ {\gamma = {{\left( {T - T_{s} } \right)} \mathord{\left/ {\vphantom {{\left( {T - T_{s} } \right)} {\left( {T_{l} - T_{s} } \right)}}} \right. \kern-0pt} {\left( {T_{l} - T_{s} } \right)}}} \hfill & {if\;T_{s} < T < T_{l} } \hfill \\ \end{array}$$

In the momentum equation, Eq. (7), the definition of the source term $\mathop S\limits^{ \to }$ is as follows^39^:

13 $$\mathop S\limits^{ \to } = - \,(1 - \gamma )^{2} \frac{{\mathop u\limits^{ \to } }}{{\gamma^{3} + \varepsilon }}C$$

In Eq. 7, $\mathop S\limits^{ \to }$ is the created porosity function, which is shown in Eq. 13 by Brent et al.^39^. C is the constant of the mushy zone morphology. The velocity is lowered to zero with a slope of C when the material solidifies. This constant ranges from $10^{4}$ to $10^{7}$ ($10^{5}$ is taken into account in this work)^40^. $\varepsilon$ is quite small (0.001) in order to avoid division by zero. A single-phase approach model with uniformly dispersed nanoparticles in the fluid was used for analysis and computations. The equations that define a nanofluid's thermophysical characteristics are shown below^11^. The density of nanofluid is given by the following equation^11^:

14 $$\rho_{nf} = \varphi \rho_{s} + \left( {1 - \varphi } \right)$$

The formula for calculating a nanofluid's heat capacity is as follows^11^:

15 $$\left( {\rho \,C_{P} } \right)_{nf} = \varphi (\rho \,C_{P} )_{s} + \left( {1 - \varphi } \right)\left( {\rho \,C_{P} } \right)_{f}$$

Brinkman's^41^ equation for calculating the viscosity is as follows:

16 $$\mu_{nf} = \mu_{f} \left( {1 - \varphi } \right)^{ - 2.5}$$

The Maxwell-Garnet equation^42^ for nanofluid thermal conductivity for nanoparticles is as follows:

17 $$k_{nf} = \frac{{[k_{s} + 2k_{f} - 2\varphi \left( {k_{f} - k_{s} } \right)}}{{\left[ {k_{s} + 2k_{f} + \varphi \left( {k_{f} - k_{s} } \right)} \right]}} \times k_{f}$$

Khanafer et al.'s equation^43^ for the nanofluid's coefficient of thermal expansion is as follows:

18 $$\beta_{nf} = \left[ {\frac{1}{{1 + \frac{{\left( {1 - \varphi } \right)\rho_{f} \beta_{f} }}{{\varphi \rho_{s} }}}}\frac{{\beta_{s} }}{{\beta_{f} }} + \frac{1}{{1 + \frac{\varphi }{1 - \varphi }\frac{{\rho_{s} }}{{\rho_{f} }}}}} \right]\beta_{f}$$

The aforementioned equations were used to calculate the thermophysical characteristics of nanofluids. According to the symbols used above, $\varphi$ represents the fluid's nanoparticle volume fraction, the subscript *nf* the nanofluid, the subscript *f *the main fluid, water, and the subscript *s* the nanoparticle.

NEPCM's thermophysical characteristics depend on the nature of the nanoparticles and the volume fraction, $\varphi$. Metal or metal oxide nanoparticles were popular additives to optimize the phase change process and increase thermal conductivity. The volume of the cylinder divided by the volume occupied by the nanoparticles is used to explain this. The modeling and equations of NEPCM, which is a combination of nanoparticles and PCM properties, are as follows^44^:

19 $$\rho_{nepcm} = \left( {1 - \varphi } \right)\rho_{pcm} + \varphi \rho_{s}$$

20 $$\left( {C_{P} \rho } \right)_{nepcm} = \left( {1 - \varphi } \right)\left( {\rho C_{P} } \right)_{pcm} + \varphi \left( {\rho C_{P} } \right)_{s}$$

21 $$\left( {q\rho } \right)_{nepcm} = \left( {1 - \varphi } \right)\left( {\rho q} \right)_{pcm}$$

22 $$k_{nepcm} = k_{pcm} \left( {\frac{{k_{s} + 2k_{pcm} - 2\varphi \left( {k_{pcm} - k_{s} } \right)}}{{k_{s} + 2k_{pcm} + \varphi \left( {k_{pcm} - k_{s} } \right)}}} \right)$$

23 $$\mu_{nepcm} = \frac{{\mu_{pcm} }}{{\left( {1 - \varphi } \right)^{2.5} }}$$

24 $$\beta_{nepcm} = \frac{{\left( {1 - \varphi } \right)\left( {\rho \beta } \right)_{pcm} + \varphi \left( {\rho \beta } \right)_{s} }}{{\rho_{nepcm} }}$$

The thermophysical characteristics of PCMs enhanced with nanomaterials are given by the aforementioned equations. For the symbols used above; $\psi$ indicates the nanoparticle volume fraction and the subscript $s$ indicates the nanoparticle. The below definitions were applied to measure the amount of energy stored in a SWH collector using PCM^45^:

25 $$E_{SWH} = (mC_{P} (T_{f} - T_{i} ))_{water}$$

26 $$E_{SWH,PCM} = (mC_{P} (T_{f} - T_{i} ))_{water} + (m\,C_{P} (T_{f} - T_{i} ) + mL)_{PCM}$$

### Boundary and initial conditions

First, the ETCSWH absorbs the sun's radiation and heats up. Simultaneously, the heat transfers from the ETCSWH tube to the HTF already existing within the ETSC tubes by convection and conduction. The PCM and the water in the storage tank are gradually heated. When the temperature of water exceeds the melting point of PCM, the melting process begins. The tube is also divided into two sections: The absorber tube's upper surface, which absorbs the average solar radiation flux of 900 W/m^2^ that shines from 11 a.m. to 3 p.m. The absorbent tube's end surface and bottom surface are considered as insulating surfaces with non-slip conditions. The ETCSWH absorber tube is positioned at a 45-degree angle, with the negative gravity pointing in the y direction. Laminar flow, transient solutions, and constant site temperatures are used in this numerical study. The simulation was performed for 4 h with the average maximum solar radiation at noon. To reduce the computational costs and simplify, the modelling of the vacuum region between the glass tubes is omitted because it has a negligible effect on the solution and is ignored^46,47^.

### Mesh structure and time step independence

**Figure 5:**
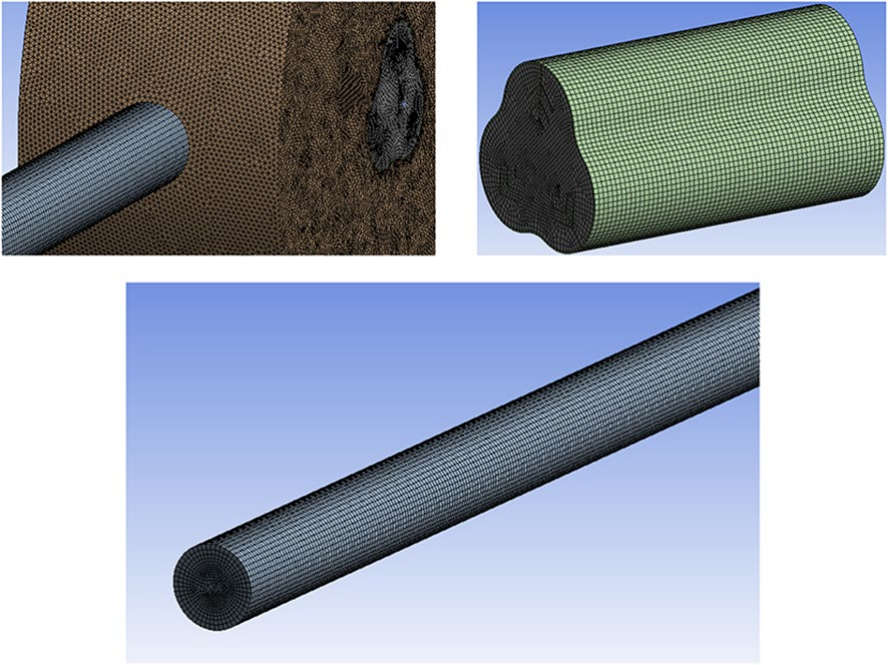
The generated mesh of an ETCSWH system.

In numerical simulation, one of the most important steps that affects the research result and increases the accuracy and confidence in the simulation is investigating the mesh quality of the geometry. In the current study, mesh independence has been evaluated by comparing liquid fraction values. The mesh of the ETCSWH system, which was created using ICEM-CFD software has been depicted in Fig. 5.

**Figure 6:**
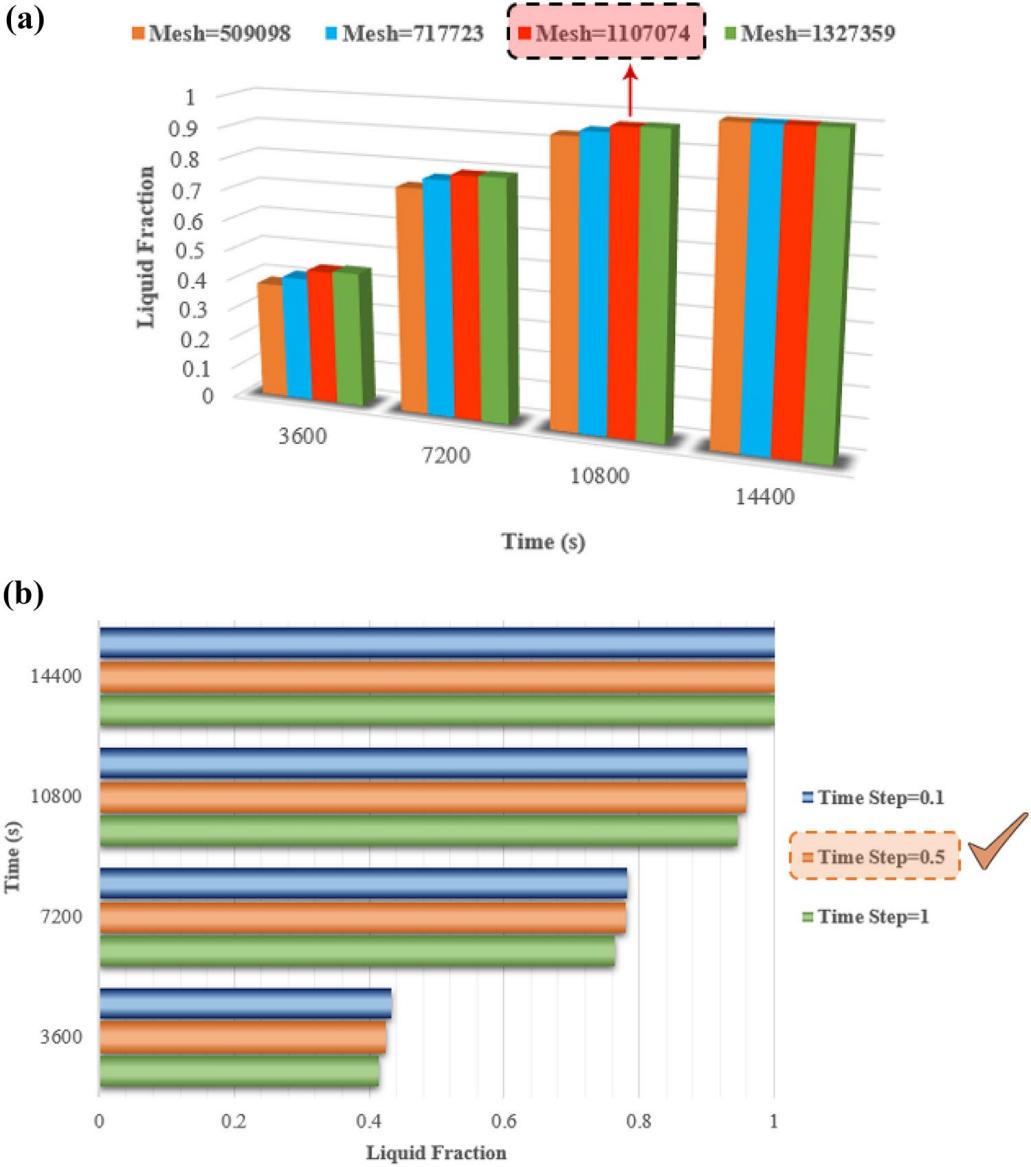
(**a**) The comparison of liquid fractions to select the best grid size. (**b**) The comparison of liquid fractions to choose the best ∆t.

The generated mesh consists of hexagonal, triangular, and tetrahedral elements. The absorber tube, PCM volume, and fin have a finer mesh. The mesh quality is good in terms of the skewness range and for most of the elements it is below 0.19. The number of tested mesh elements varied from 500,000 to 1,500,000. According to the test results for 4 h of simulation, the best case with a very low error with a total number of 1,107,074 elements is shown in Fig. 6a. It is significant to select the proper time step size for the transient simulation process in order to reduce computation time and obtain accurate results. The time step analysis has been performed, taking the melting fraction into account for the most complex case during the four-hour simulation. As inferred from the results, to achieve time step independence, 3 alternative time steps of 0.1, 0.5, and 1 s were examined. As depicted in Fig. 6a,b time step of 0.5 s is suitable for simulation.

## Results and discussion

In this research, we want to learn more about improving heat transfer in an ETCSWH system. A unique PCM chamber is utilized inside the water storage tank to store thermal energy. Heat transfer has been improved using NEPCM. NEPCM is obtained from the combination of 3% volume fraction of Al_2_O_3_ nanoparticles with PCM. Aluminum oxide is preferred because of its benefits such as high heat conductivity, which speeds up PCM melting, a relatively, which prevents sedimentation, and cost-effectiveness compared to other selected nanoparticles. The simulation process was carried out for 4 h, with most of the results presented for 1, 2, 3 and 4 h. To increase heat transfer and accelerate the melting process, nanofluid (water + 3% Cu) and fins are also used for the PCM enclosure. In this study, 8 different cases shown in Table 2 have been examined. In the following, we discuss the results of this simulation, which are given in the next sections.

**Table 2:** Various computational cases.

Case	Suggested system
1	Case PCM
2	Case Nanofluid
3	Case NEPCM
4	Case NEPCM + Nanofluid
5	Case PCM + Fin Tank PCM
6	Case Nanofluid + Fin Tank PCM
7	Case NEPCM + Fin Tank NEPCM
8	Case NEPCM + Nanofluid + Fin Tank NEPCM

### Examining the system's temperature and melting process both with and without fins

**Figure 7:**
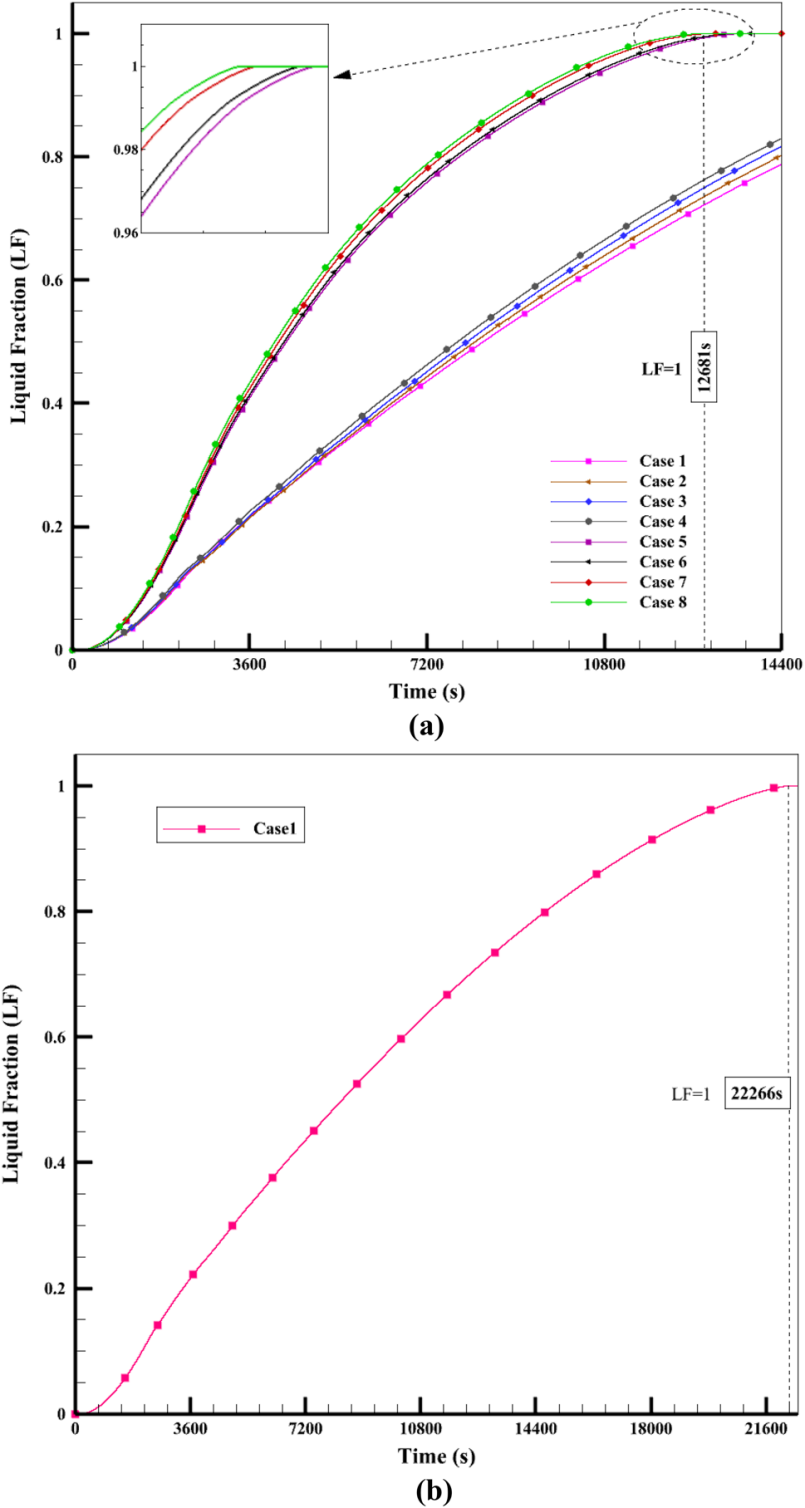
(**a**) The comparison of the melting fraction amounts under various melting cases, (**b**) Complete melting of the Case 1.

In this section of the present study, we investigate the PCM's charging process within the ETCSWH tank. Several techniques, such as nanofluid, NEPCM, and the insertion of tree-type fins in the PCM chamber, were used to manage the charging process, enhance heat transfer, and accelerate the PCM melting in the tank. Three tree-type copper fins with equal dimensions are placed inside the PCM chamber. To simulate the melting process, a maximum average flux of 900 W/m2 was applied for 4 h at a 45-degree angle to the ETC absorber. During the simulation, the results for several scenarios were compared as diagrams and contours of the melting fraction and NEPCM's average temperature. Figure 7a shows the change in melting fraction for 8 cases during 14,400 s (4 h of charging time) and Fig. 7b shows the complete melting of Case 1.

**Figure 8:**
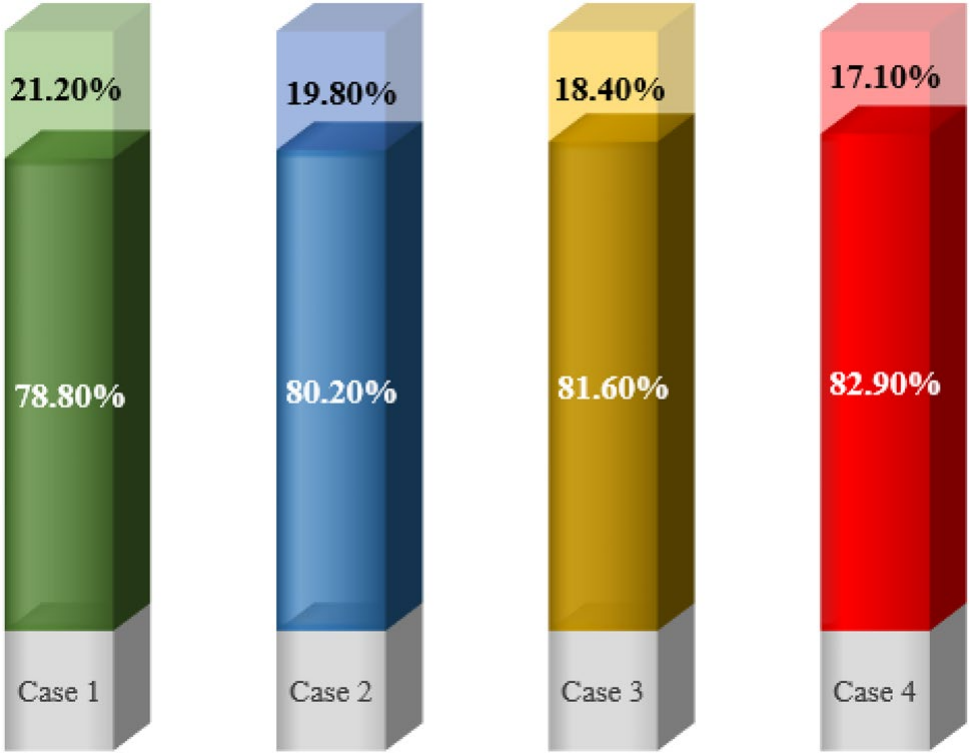
The melting percentage of the first 4 cases in a period of 4 h.

As it is clear from Fig. 7, the cases without fins did not reach complete melting within 4 h, while the cases with fins reached complete melting earlier than the simulation time. And they saved more energy than cases without fins. Case 1 had the lowest amount of melting during the simulation period and reached LF = 1 in 22,266 s, which can be seen in Fig. 7b and from the liquid fraction diagram (Fig. 7a) it is clear that the amount of melting in the ECTSWH system is respectively using nanofluid, NEPCM and the simultaneous combination of nanofluid and NEPCM is improved. The value of the percentage melting of cases without fins is given in Fig. 8. For example, in Case 1, 78.8% of it was melted and 21.2% was not melted during the simulation period. Case 4 with nanofluid and NEPCM has improved by 5.2% compared to simple Case 1. PCM's inadequate thermal conductivity, which slows down the liquid process, is the cause of Case 1's sluggish melting process. However, by using nanofluid and NEPCM, the heat transfer is accelerated, and that issue is more effective in cases with fins.

**Figure 9:**
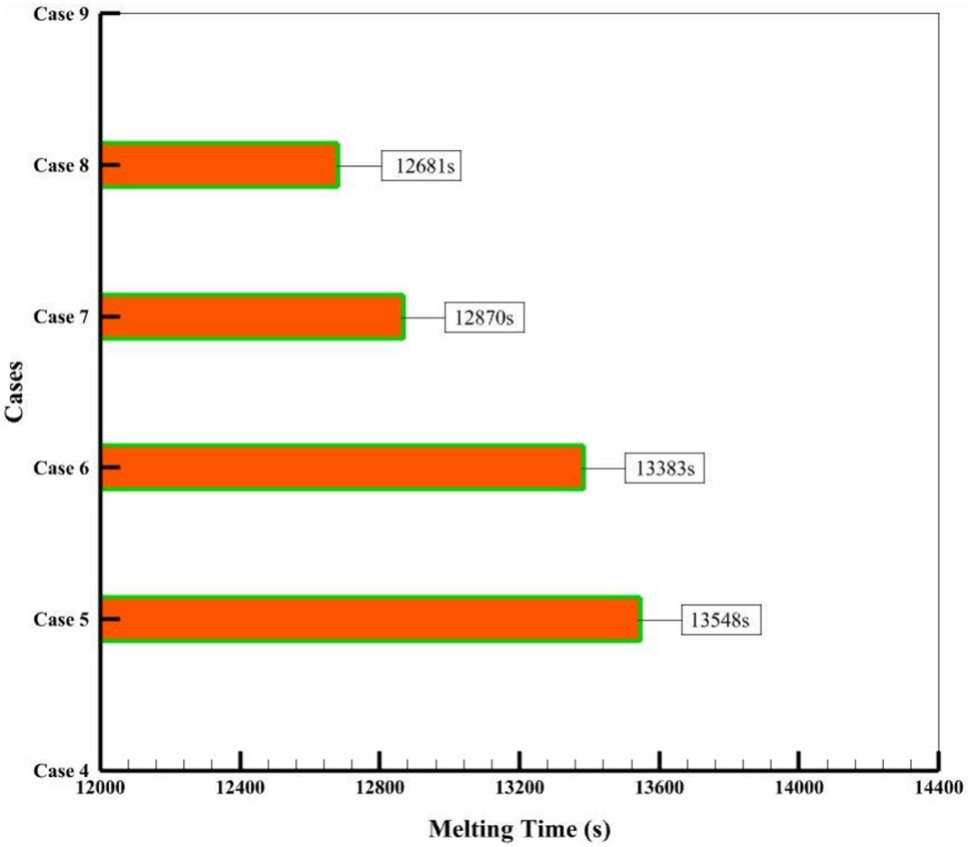
The comparison of the respective melting times for several fin-equipped system cases.

Figure 9 shows that the 8-fin case with nanofluid and NEPCM is the best case, which is completely melted in 12,681 s (equivalent to approximately 3.52 h), earlier than the other cases, and the liquid fraction reaches 1. A comparison of complete melting times for finned cases is shown in Fig. 9. Case 8 reaches complete melting 867 s (almost 15 min) earlier than Case 5. In the period of 12,681 s when Case 8 is completely melted, Case 1 has melted 71.58% of its value. Therefore, Case 8 has improved by 39.7% in contrast to Case 1. Additionally, Case 1 reaches complete melting after 22,266 s (equivalent to 6.185 h), which is delayed by 9585 s (equivalent to 2.67 h) compared to Case 8. The collected data indicate that the suggested solutions significantly enhance heat transfer, which causes more PCM to melt in the same period of time.

**Figure 10:**
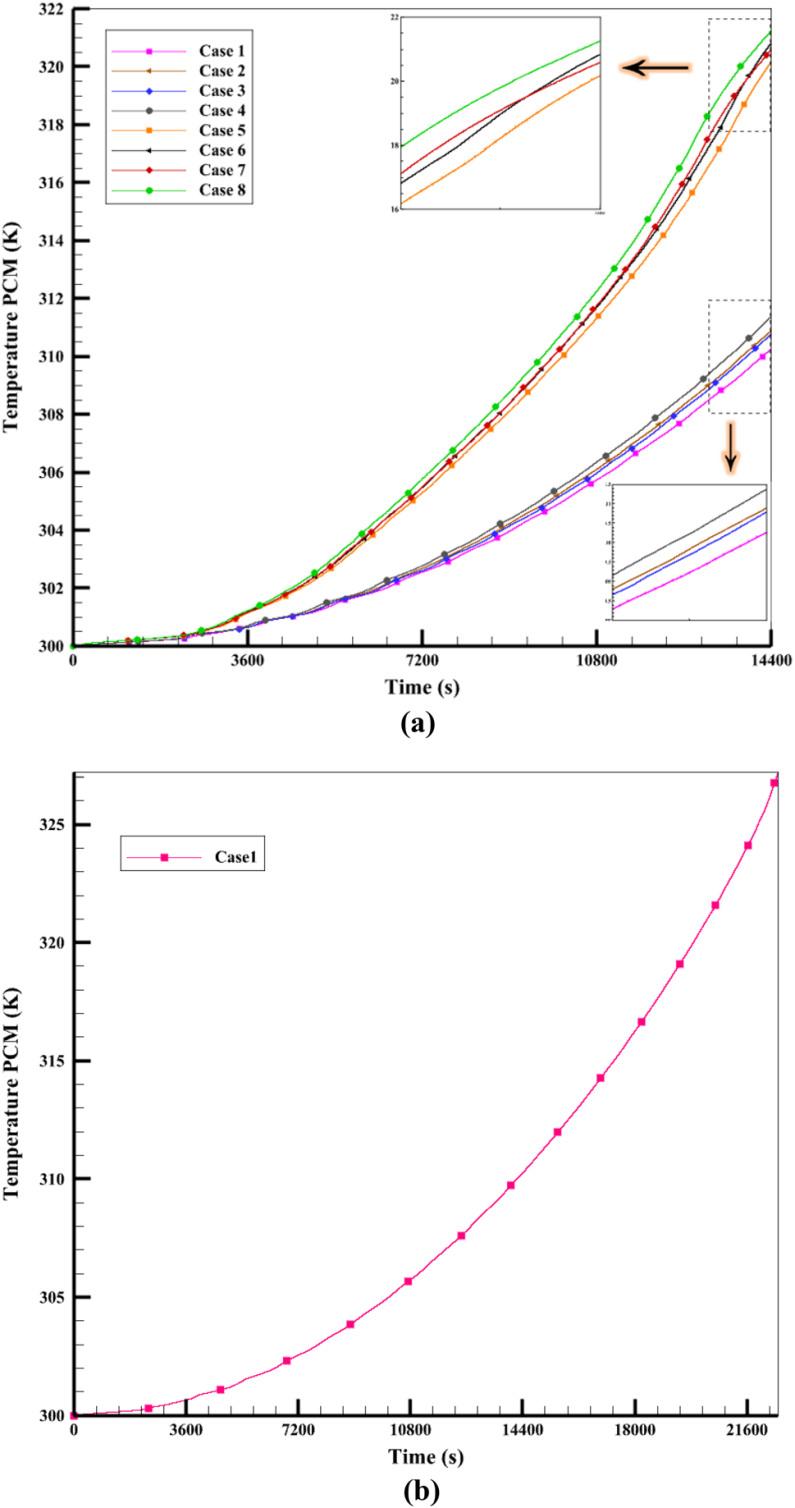
(**a**) The comparison of the T_PCM_ over 4 h for various cases, (**b**) Average PCM temperature of Case 1.

Figure 10a displays the T_PCM_ for different cases during the time period of 14,400 s, and Fig. 10b illustrates the PCM temperature diagram for Case 1. As mentioned earlier, the use of nanofluid, NEPCM, and fins makes the average temperature of NEPCM (321.25 K) in Case 8 the highest among the other cases, which indicates that this case can produce more heat than the other cases in the same way. The lowest PCM temperature (310.26 K) belongs to Case 1, which absorbs the least energy, has the least heat transfer, and has the lowest heat transfer. The graphs in Fig. 10 show that the cases with NEPCM and nanofluid are very close to each other most of the time, but the simple case and the case with NEPCM and nanofluid have a bigger difference, which is due to the use of NEPCM and nanofluid that enhance heat transfer and absorb more energy (higher temperature). Also, cases with fins have a faster temperature increase than cases without fins. Based on the mentioned information, the amount of energy absorption and the T_PCM_ in Case 8 show that this case had the best performance.

**Figure 11:**
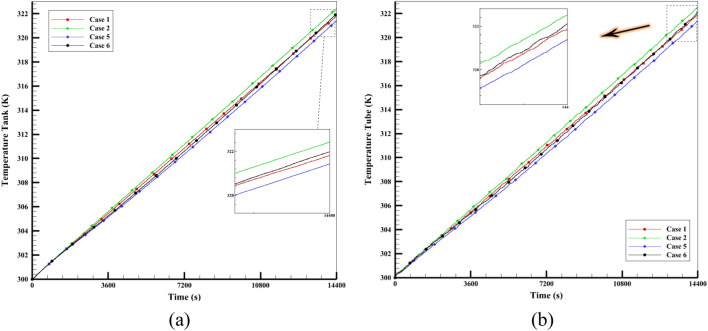
The average fluid temperature in a period of 4 h inside (**a**) tank (**b**) tube.

Figure 11 displays the average temperature of the fluid in four cases (one with and one without fins and one with and one without nanofluid) inside the tube and storage tank. The rate at which the tank's fluid temperature rises in the first 40 min of flow time is almost the same in all four cases. But when the melting process begins, the average temperature of the HTF changes by 4 to 5 degrees. The slope of the liquid fraction graphs and the temperature of the fluid for each of the four cases change as the melting process continues. After one hour of the operation, the average fluid temperature difference between Cases 2 and 5 increases and reaches its maximum value. Case 2 without fins, because it has nanofluid, has a higher fluid temperature inside its tank than the other 3 cases. Similarly, in Case 5, even though it has water fluid, the PCM chamber has fins. Case 5 with water and fin fluid has a lower fluid temperature than the other 3 cases because it needs more heat to melt the PCM.

The tank temperature in Case 1 without fins and nanofluid is almost the same as in Case 6 with fins and nanofluid as shown in Fig. 11a. This is because Case 6 has a nanofluid, which makes the fin absorb more heat from the PCM and lowers the storage tank temperature (similar to Case 1). Figure 11b shows the same explanations. Based on the information and the data from Fig. 11a, Case 5 has a lower average fluid temperature than the other three cases. This is because Case 5 has a higher heat transfer rate (since it has fins) and more heat absorption by the PCM. The PCM gets this energy from the HTF inside the tank, which makes the fluid temperature lower than in the other 3 cases. The results show that the difference in heat transfer among the paraffin chamber and the surrounding fluid causes a difference in the liquid fraction and the average temperature in different cases. Adding fins to the PCM chamber also makes the PCM melt faster and reduces the fluid's average temperature in the tank until the PCM is fully melted. Therefore, choosing a PCM case with fins to save energy and provide hot water at night is better than a case without fins.

**Figure 12:**
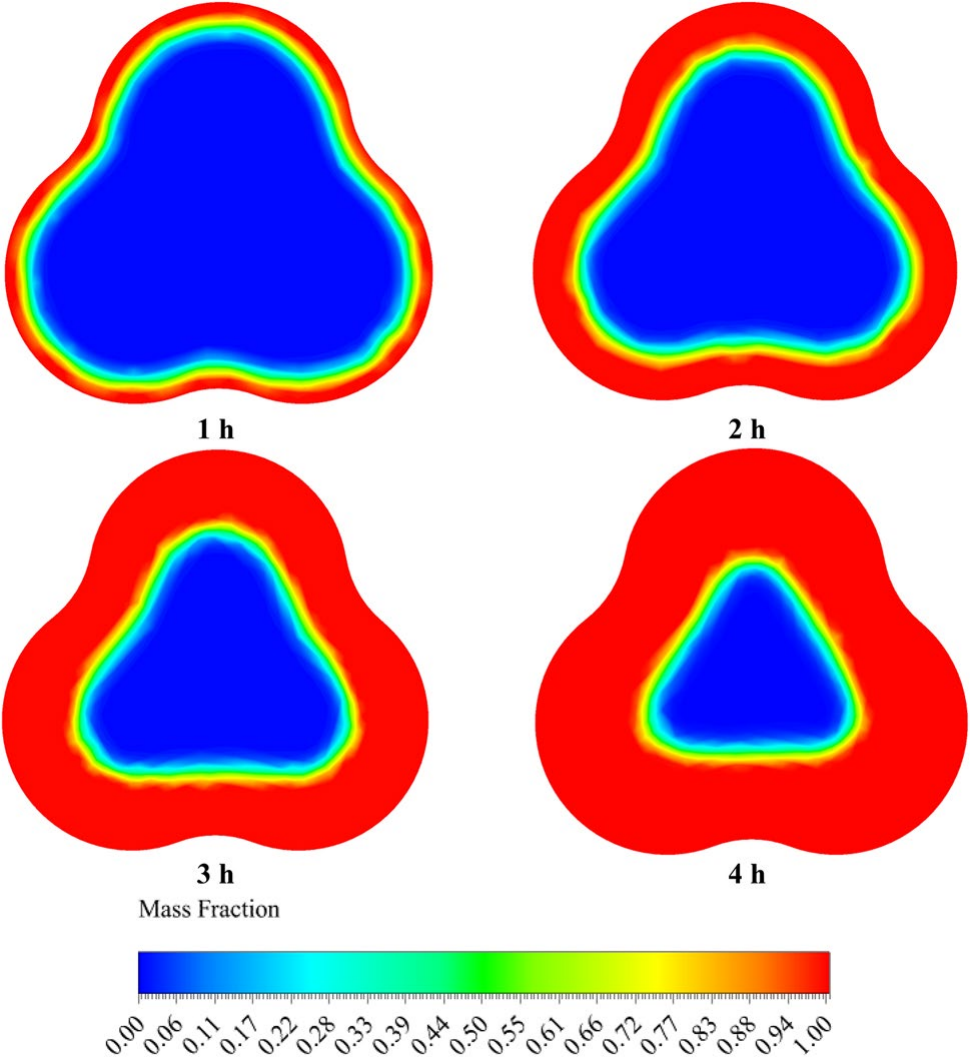
The comparison of LF for Case 1 at various time periods.

**Figure 13:**
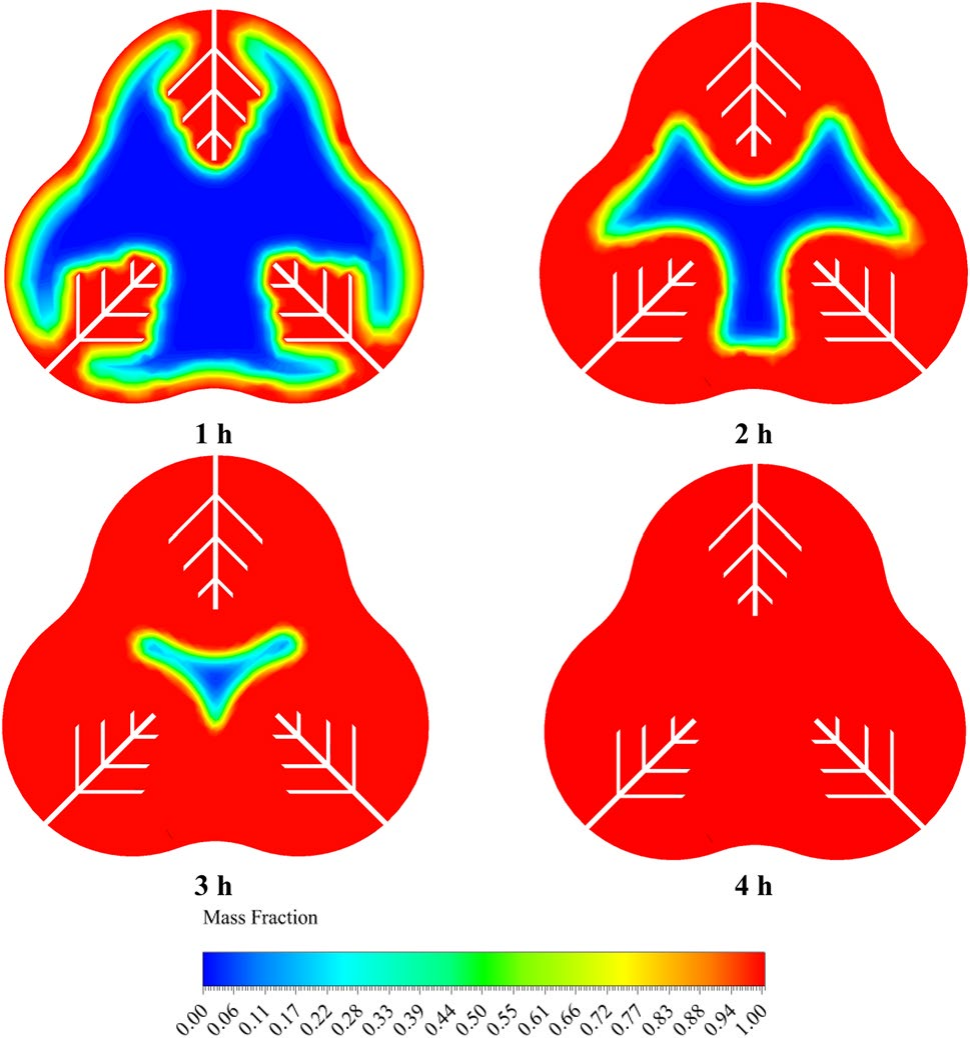
The analogy of the liquid fraction for Case 8 at various time intervals.

Figures 12 and 13 compare the melt fraction contour for the middle section cut from the energy storage tank for Case 1 without fins and Case 8 with fins at different times. The melting always starts at the inner radius of the chamber. However, the temperature difference created inside the tank by free displacement flow makes the upper part of the tank hotter, and the PCM in the upper half of the chamber melts faster than the lower part. Also, the cases without fins shown in Fig. 12 do not reach complete melting at 4 h. But the cases with fins (Fig. 13) reach full melting before 4 h. In Case 1, 21.2% of the PCM remains solid, and at 12,681 s, when Case 8 is fully melted, Case 1 only melts 71.58% of the PCM.

**Figure 14:**
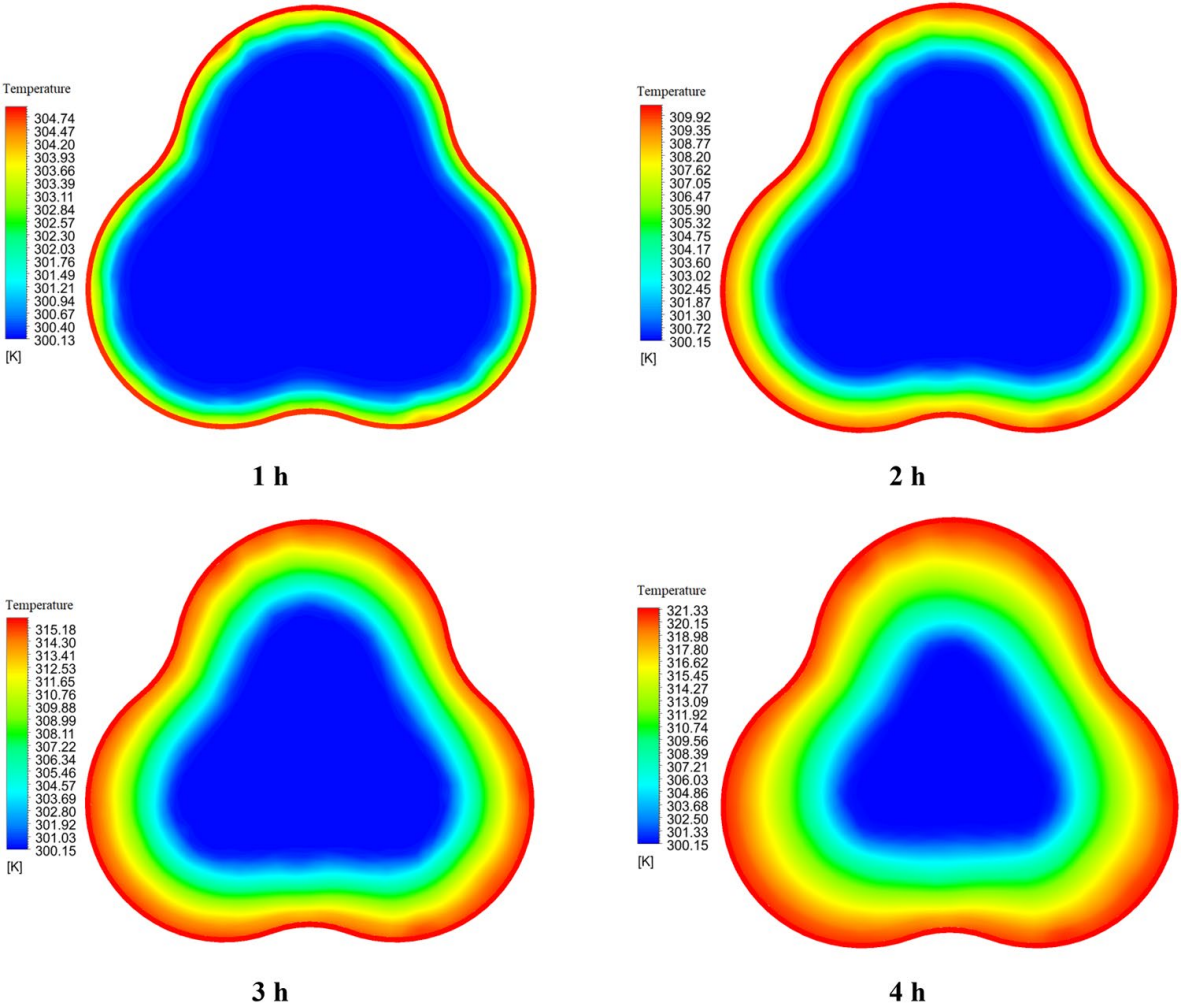
The comparison of PCM temperature contour at various times for Case 1.

**Figure 15:**
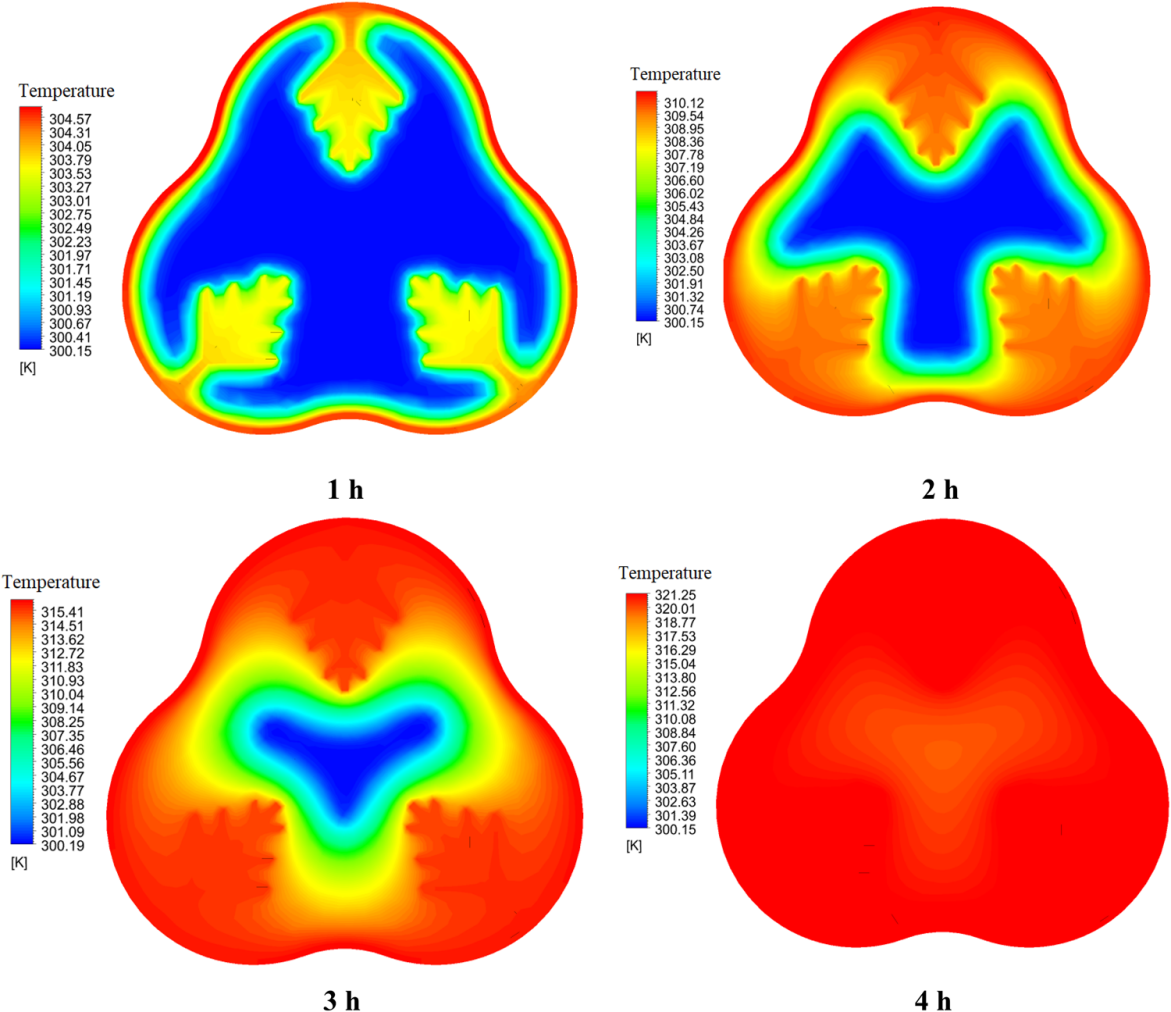
The comparison of PCM temperature contour at various times for Case 8.

Figures 14 and 15 show the temperature contour of the PCM for Case 1 and Case 8 at different times. The contours obtained in this study are from CFD-POST software. To better display the temperature contour from the local mode of the software, the middle section of the PCM chamber is used for the contour. Near the wall of the PCM chamber, the color is red because it is in contact with the hot fluid inside the tank, and the legend has its own temperature for each hour. As we move towards the center of the PCM chamber, the temperature distribution gradually decreases and the blue area is solid PCM. Figure 14, like Fig. 12, shows that the PCM is not fully melted and the blue solid area has a temperature of 300.15 K (PCM solid temperature). The same explanation applies to Fig. 15. But in this case, the PCM is completely melted, and its temperature contour is uniform at 4 h, which confirms this.

### The efficacy of adding fins and nanomaterial

This work models the melting using the porosity enthalpy approach, and the nano-powders in the water-base fluid using the single-phase model^11,39^. Cu nanoparticle (with a volume fraction of 3%), which has better performance than other nanoparticles in increasing the performance of solar systems (for nanofluid)^10,48,49^, is used. Additionally, 3% volume fraction aluminium oxide nanoparticles are used to accelerate phase change heat transfer. Based on the good results obtained from the studies^50–53^, Al_2_O_3_ is chosen as a nanoparticle for PCM composition. The average reservoir temperature is compared as a factor to evaluate the thermal treatment of the system for nanofluids. Case 1 without nanofluid and Case 2 with nanofluid (or Case 5 and Case 6) are compared. Figure 11a shows the fluid's average temperature inside the storage tank. The graph shows that Case 2 with a fraction of 3% Cu nanoparticles added to the water leads to more heat transfer to the reservoir and a higher average reservoir temperature than Case 1. The same process happens in the absorber tube shown in Fig. 11b. The tank containing nanofluid has a higher average temperature as a result of the simulation. The viscosity of the nanofluid reduces as the temperature rises, increasing the fluid flow rate. The research shows that PCM heats up more when Al_2_O_3_ nanoparticles are added than when PCM is used alone. Adding 3% Al_2_O_3_ nanoparticles to paraffin wax increases heat transfer and stores more energy than pure PCM. It also makes it possible to recover the stored energy quicker at night. Figure 7 illustrates that the melting fraction diagram for Case 3 with NEPCM is greater than the diagram for Case 1 with PCM. The viscosity of the nanofluid also reduces as the temperature rises, increasing the fluid flow rate. The research shows that PCM heats up more when Al_2_O_3_ nanoparticles are added than when PCM is used alone. Adding 3% Al_2_O_3_ nanoparticles to paraffin wax increases heat transfer and stores more energy than pure PCM. It also makes it possible to recover the stored energy faster at night. Figure 10 shows that Case 3's melting temperature is greater due to the combination of Al_2_O_3_ nanoparticles with PCM, which increases heat transfer. The same analysis applies to Cases 5 and 7. Compared to Case 5 with fins without NEPCM, Case 7 with finned NEPCM has a 5% shorter melting time due to the addition of Al_2_O_3_ nanoparticles. It also improves the melting process of Case 3 with NEPCM by 3.6% compared to Case 1 with PCM. As a result, more heat is stored while charging due to the incorporation of NEPCM inside the ETCSWH system. Figure 7 illustrates that the melting fraction diagram for Case 3 with NEPCM is greater than the diagram for Case 1 with PCM.

Table 3 compares the average temperatures of pure water and nanofluid at different times with those of the storage tank in the finless cases. The table shows that employing a 3% volumetric fraction of Cu nanoparticles compared to pure water at the same time improves the temperature. Every hour, the average temperature of water increases approximately 5.47 K, and the average temperature of nanofluid increases approximately 5.61 K. This means that heat transfer is augmented, and the merit of using particles for productivity can be seen.

**Table 3:** During the simulation period for the finless case, the water and water + 0.03%Cu nanoparticle average temperatures in the tank and tube.

Testing fluid	$\varphi$	Time (h)	$T_{ave}$ Tank	$T_{ave}$ Tube
Water	0	1	305.4491	305.4591
		2	310.8619	310.9613
		3	316.3288	316.3748
		4	321.8143	321.882
Water + Cu	0.03	1	305.6036	305.6763
		2	311.171	311.2841
		3	316.7852	316.8578
		4	322.4253	322.5097

Table 4 compares the HTF temperatures in the storage tank of finned cases and the tube filled with water and nanofluid at various times. The table shows that the efficiency of the ETCSWH s improved by the presence of Cu particles with a 3% volumetric fraction compared to the base fluid, even for finned cases. The cases in Table 4 have fins that enhance heat transfer within the PCM chamber, which lowers the temperature of the fluid inside the storage tank and tube than the temperature of the HTF in Table 3. which explains the lower average fluid temperature in different hours in Table 4 than in Table 3. The average water temperatures of the storage tank and tube increase by approximately 5.6 K, and the average temperature of the nanofluid increases by approximately 5.8 K per hour. This demonstrates the merit of using Cu nanoparticles in the water-base fluid for the system performance.

**Table 4:** During the simulated period for the cases with fins, the water and water + 0.03% Cu nanoparticle average temperatures in the tank and tube.

Working fluid	$\varphi$	Time (h)	$T_{ave}$ Tank	$T_{ave}$ Tube
Water	0	1	305.1571	305.1079
		2	310.3631	310.3359
		3	315.8269	315.7497
		4	321.4285	321.3983
Water + Cu	0.03	1	305.262	305.3088
		2	310.604	310.6926
		3	316.2095	316.3184
		4	321.9836	322.1223

**Figure 16:**
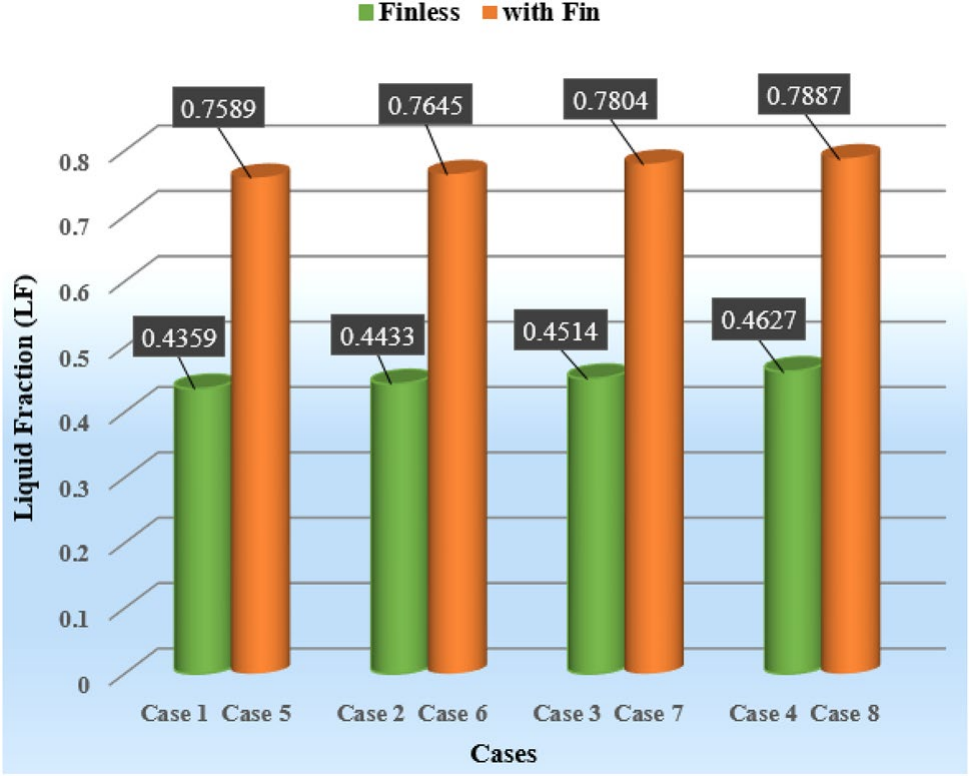
The melting amount of 8 cases after 2 h (with fin and without fin).

This simulation mainly investigates how the efficiency of the ETCSWH system is intensified by the addition of copper fins in the shape of tree-type in the PCM chamber. There are 3 fins of the tree type with variable branches, whose length dimensions are 4, 8, 12 mm from small to large, respectively, its thickness is 1 mm, and the spacing between the branches is 8 mm. This study equalized the volumes of the geometries with and without fins by trial and error, and selected the best geometry among different options. Figure 16 compares the PCM melting rates for all cases with and without fins after two hours of simulation. The results clearly demonstrate that using fins in the PCM chamber enhances heat transfer, increases melting, and speeds up the melting process. In addition, Case 8 with fin has the maximum amount of liquid fraction of 0.7887 after 2 h of simulation, which is 70.46% more than the case without fin (Case 4). Case 8 also has nanofluid, NEPCM, and fins, which give it the best performance, the fastest melting time and the largest amount of melting compared to other cases.

Table 5 compares T and LF at various times for the case with PCM and the case LF for the case with NEPCM in the finless geometry (Cases 1 and 3). The effect of a 3% volume fraction of Al_2_O_3_ nanoparticles compared to pure PCM was also investigated. The table shows that the use of Al_2_O_3_ nanoparticles improves heat transfer and melting processes at every hour of simulation. The use of nanoparticles to increase performance is obvious.

**Table 5:** During the simulation period for the finless cases, the average temperature and melting amount of PCM and PCM + 0.03% Al_2_O_3._

Material	$\varphi$	Time (h)	$T_{ave}$ PCM	LF
PCM	0	1	300.6428	0.215949
		2	302.5588	0.4359
		3	305.7404	0.62771
		4	310.2605	0.788149
PCM + 3%Al_2_O_3_	0.03	1	300.6544	0.217381
		2	302.6084	0.4514
		3	305.9746	0.652604
		4	310.7857	0.816778

Table 6 compares T and LF in the fin geometry with the average temperature of NEPCM and LF at different times (Cases 5 and 7). The table shows that the presence of Al_2_O_3_ nanoparticles with a volume fraction of 3% increases the heat transfer and improves the melting process compared to pure PCM, even for finned cases. Cases with nanoparticles have a greater LF and average PCM temperature, which accelerates the melting process and stores more energy. This demonstrates that the use of Al_2_O_3_ nanoparticles is beneficial. Comparing Tables 5 and 6, it can be seen that Table 6, which is data related to cases with fins, has better range of numbers than Table 5.

**Table 6:** During the simulation period for the cases with fins, the average temperature and melting amount of PCM and PCM + 0.03% Al_2_O_3_.

Material	$\varphi$	Time (h)	$T_{ave}$ PCM	LF
PCM	0	1	301.1426	0.40934
		2	305.2921	0.7589
		3	311.3272	0.940122
		4	320.1744	1
PCM + 3%Al_2_O_3_	0.03	1	301.167	0.424057
		2	305.4842	0.7804
		3	311.7585	0.958543
		4	320.5861	1

### Velocity distribution and temperature contour for nanofluid in tube and storage tank

**Figure 17:**
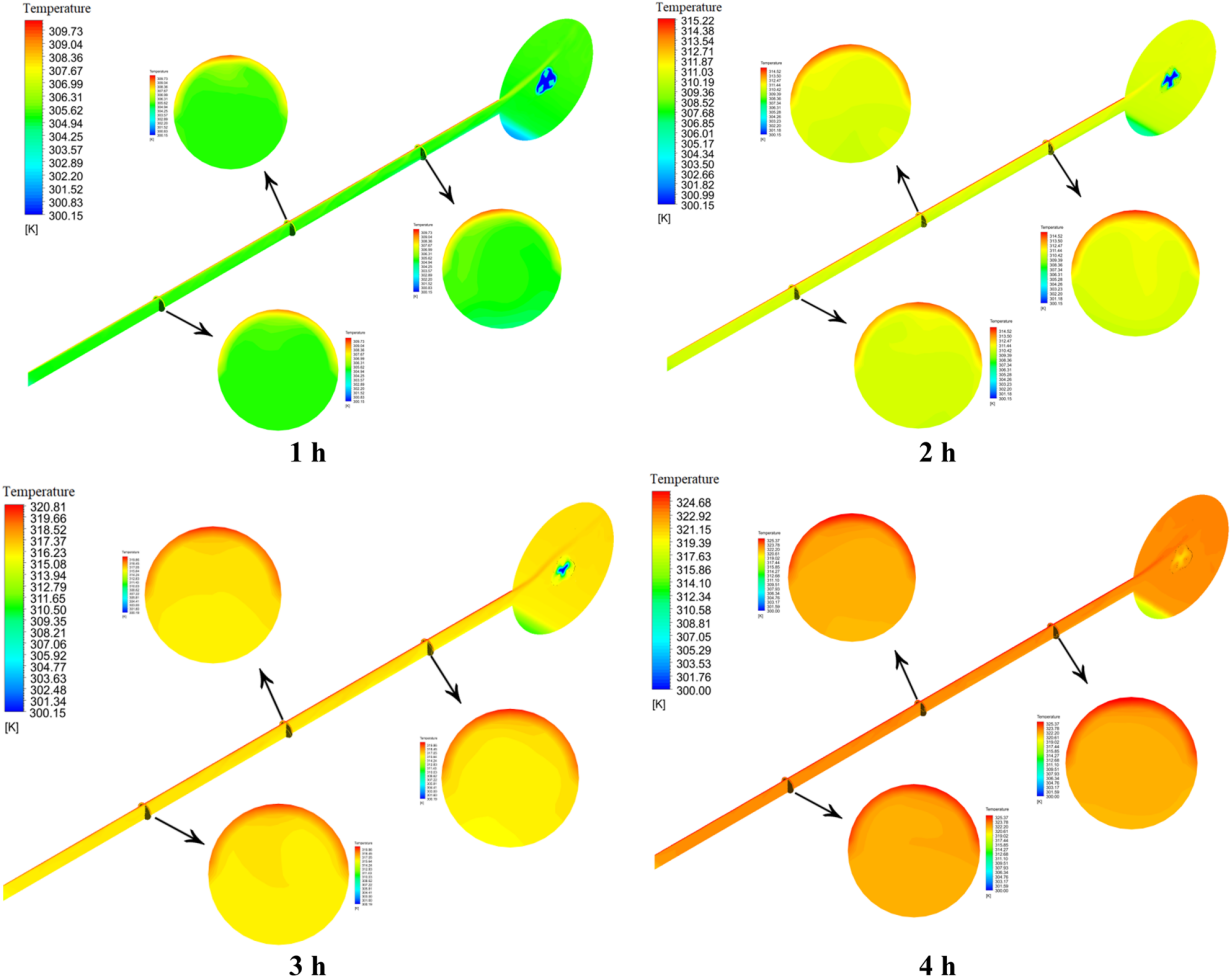
The temperature contour for the copper/water nanofluid during the simulation times.

This section analyzes the behavior and fluid flow in the ETCSWH system. It consists of two parts: the first part examines the results related to temperature contour and the velocity distribution for the absorber tube, and the second part does the same for the storage tank. Three vertical sections were selected along the absorber tube's beginning, middle, and end to closely analyze the circulation behavior of the nanofluid during natural convection inside the ETC absorber tube. The distance of these three planes from the end of the tube is 1000, 500 and 1500 mm, respectively. The process of natural flow circulation inside the ETCSWH system is as follows. Despite its increased density and the pull of gravity, the cold fluid initially enters the tube from the bottom part of the storage tank and flows towards the tube's closed end. After the HTF inside the absorber tube absorbs the heat from solar energy shining on its top surface, the natural convection process starts. Specifically, natural convection is caused by a difference in fluid temperatures. The fluid in the top part of the tube is hotter because the incoming flux radiates to the upper surface. The fluid's density decreases and a buoyancy effect is generated when it heats up in the top part of the tube. As a consequence, the heated fluid circulates back to the storage tank by using a natural convection flow that is created from the upper part of the absorber, and this circulation process continues. Figure 17 shows the temperature contour of the entire ETCSWH system and three vertical sections in different parts of the tube corresponding to Case 8 at different hours. The nanofluid is located in the top half of tube, whose upper surface is exposed to sunlight, as seen from the shape of the sections cut in the tube. The temperature is higher there. The temperature of the nanofluid in the tube's bottom half, which it passes through as it flows from the storage tank to the tube's closed end, is lower. The storage tank experiences the same phenomenon. The heated fluid from the top part returns from the tube to the storage tank due to natural displacement and warms the storage tank's top part. However, in the storage tank's bottom part, natural convection does not occur, so it remains in the stagnant (stillness) region at a lower temperature.

**Figure 18:**
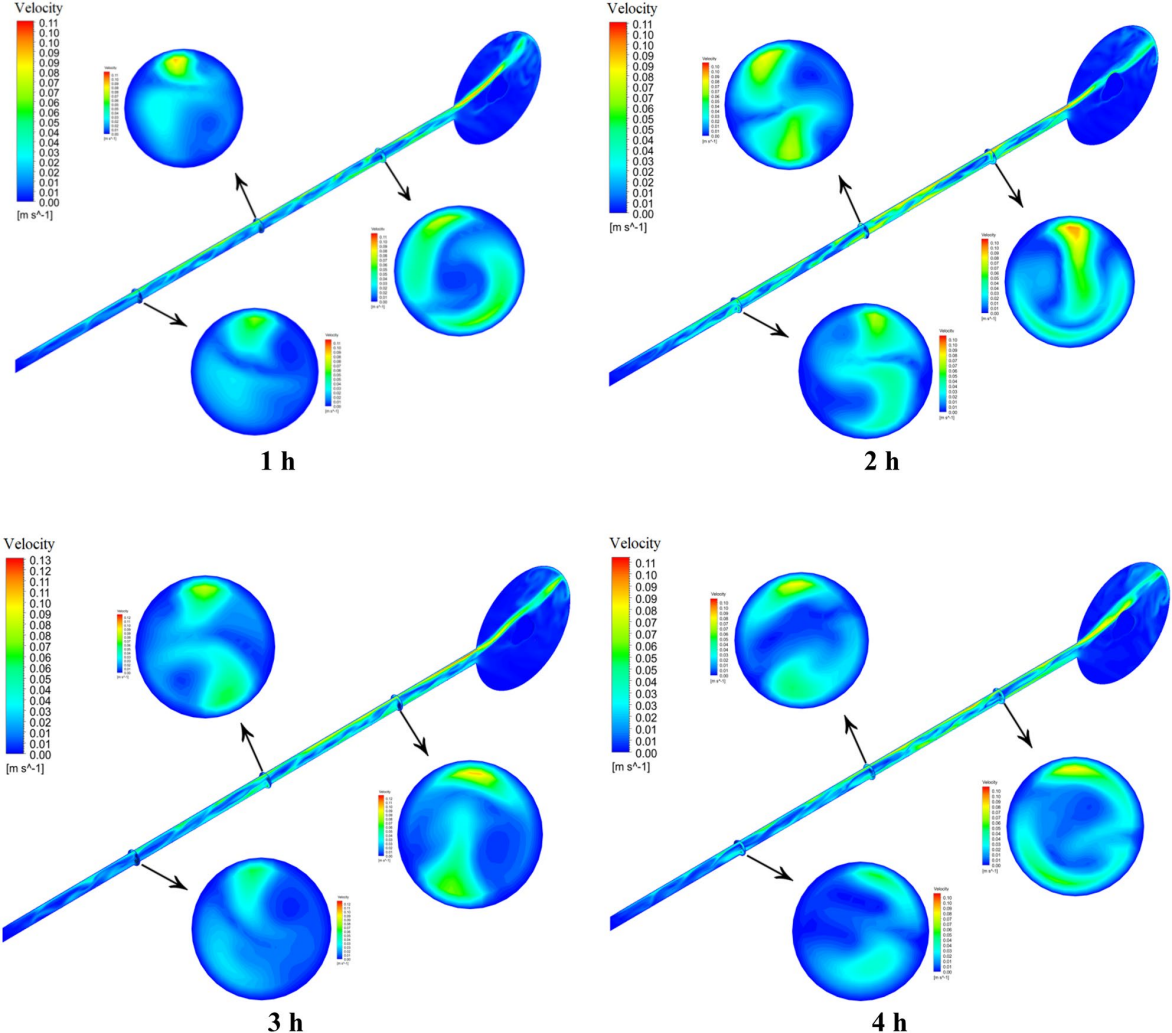
The velocity contour for the copper/water nanofluid during the simulation times.

Figure 18 displays the nanofluid velocity contour for Case 8 at each hour of simulation for the entire ETCSWH system and the three cross-sections taken in the tube. The cold fluid enters the tube and flows to the lowest point of the system (the tube's closed end). The velocity is greater in the top part of the absorber tube than in other adjacent parts, as seen from the velocity contour of various parts of the absorber tube at various times. This is because the return flow of nanofluid to the storage tank is driven by natural convection. In this simulation, we examined three sections. The legend related to the velocity contours in each hour shows the velocity of that area. The maximum velocity value in each section in the fourth hour for Sects. 500, 1000 and 1500 mm from the closed end of the tube is 68.8, 98.7 and 10.7 mm/s, respectively, and the tube's maximum temperature value is around 24 K. It is necessary to mention that the velocity at different times (other hours) and at different points can be lower or higher than these values. Moreover, the maximum velocity achieves its peak value of 11.41 mm/s when the fluid enters the storage tank at the open end of the absorber (the area 1800 mm from the closed end of the tube). The buoyancy force acting in the tube's circumferential direction and towards the top half of the tube makes a part of the input flow (from the storage tank to the tube) divert into the secondary flow, according to the contours. The velocity of the flowing nanofluid decreases to the lowest point of the tube, as shown by the cross-sections. It declines as a result of the friction's influence on the boundary layer, which makes it thicker. At the tube's closed end, there is a stationary region with no velocity, where natural convection does not occur, between the two upper and lower layers that flow in opposite directions. A sliding layer is formed between them due to the friction between fluid particles.

**Figure 19:**
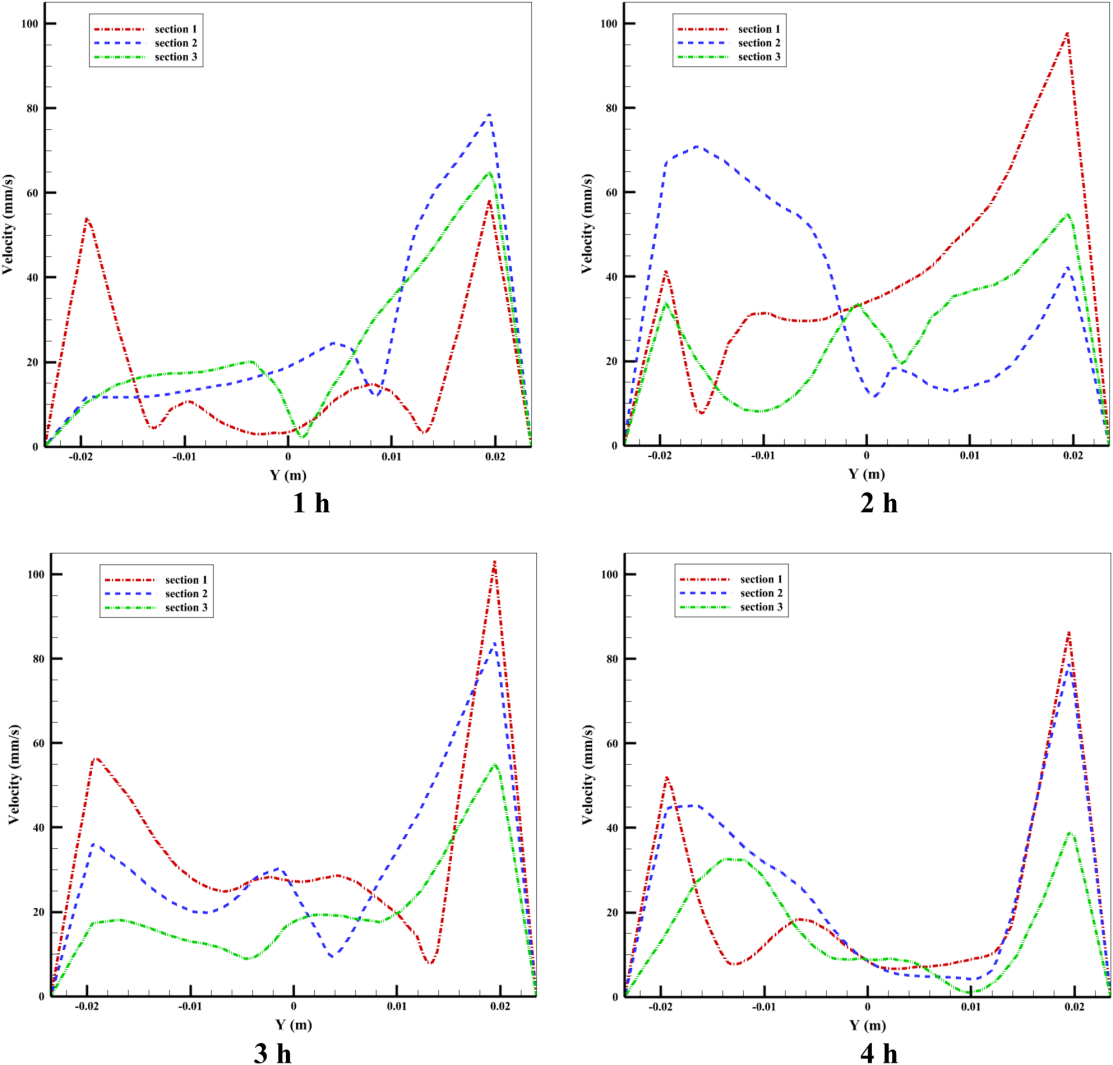
The distribution of velocity at various tube sections.

**Figure 20:**
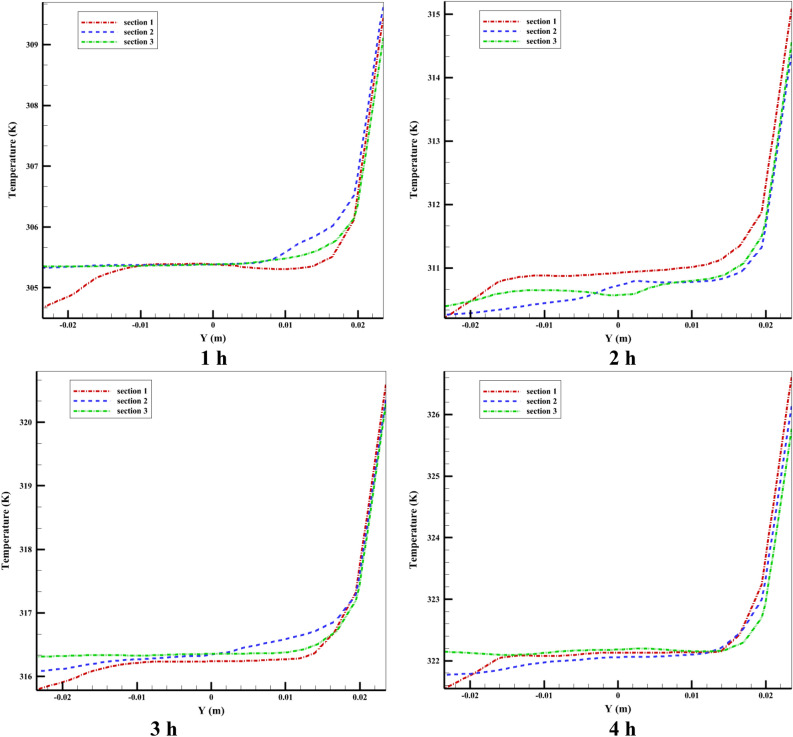
The distribution of temperature at various tube sections.

The velocity distribution is shown in Figs. 19 and 20 depicts the fluid temperature distribution in the radial direction (y = − 0.0235 − 0.0235) of the tube in three different sections (lines) for Case 8 at different times. It should be noted that in positive y's we have negative velocity, because in the graph, the value of velocity is shown as an absolute value. It is noteworthy that the velocity value is positive and negative in y and varies in the tube's top half, and the velocity value in negative y is positive and varies in the tube's lower half. It is also evident from the figure, the right side of the graph (positive y) which has a higher velocity value corresponds to the velocity of the fluid in the upper half of the tube, which is hotter, faster, less dense, and has a natural convection flow from the tube to the storage tank. Cold fluid is moved from the tube's bottom half to the closed end of the absorber tube (inlet flow) when the velocity is positive and heated fluid is moved from the tube's top half to the storage tank when the velocity is negative. As it is clear from the figure, the velocity in the center of the tube is not zero, this happens because, near the shear layer, hot fluid leaves the flow towards the storage tank for reheating and returns to the cold fluid in the lower half of the tube. It is worth mentioning that the graphs drawn are the velocity values at the points on a line in the radial direction, which can be different at different times and other radial directions of these values.

As we know, the velocity at the closed end of the tube decreases because it reaches the area where natural flow does not occur (stationary area). The lowest velocity is related to the last Sect. 3 near the quiescent area of the tube. Figure 20 shows the temperature distribution for Case 8 at various hours in three vertical sections along the tube's radial direction. The figure illustrates this: the HTF flow from the storage tank to the tube (input flow) has a lower temperature than the HTF flow from the tube to the storage tank (output flow). For all three sections during the simulation, the graphs were almost identical. The most significant finding from the graphs is that there is a substantial increase in temperature from y = 0.012 to the top surface, as seen in the figure. This can be explained when a part of the inlet flow joins the secondary flow due to the buoyancy force in the tube's circumferential direction and towards the top part of the tube, which increases its temperature. In the following part of this section, we will examine the velocity distribution and temperature contour inside the storage tank.

**Figure 21:**
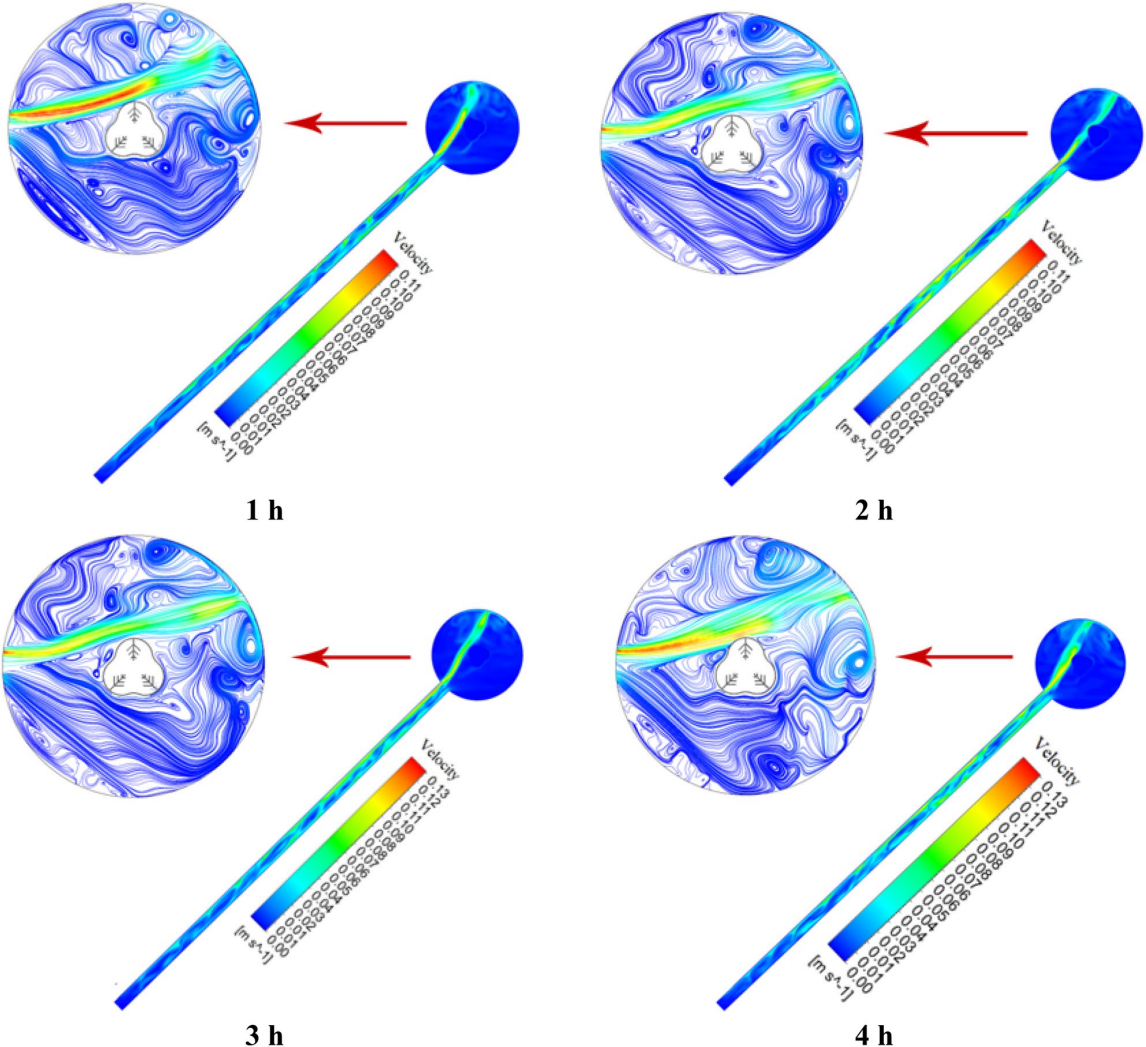
The nanofluid velocity distribution at different times.

Figure 21 shows the velocity flow lines inside the storage tank for Case 8 using nanofluid. The structure of the fluid flow and the formation pattern of the natural convection flow can be seen in this Figure. As it is evident from the velocity contour inside the storage tank, the fluid has a higher speed when entering the storage tank than other places. The hot and cold fluid layers are separated inside the absorber tube, which is divided into two halves. The hot fluid is located at the top of the absorber tube, while the cool fluid is located at the bottom.

**Figure 22:**
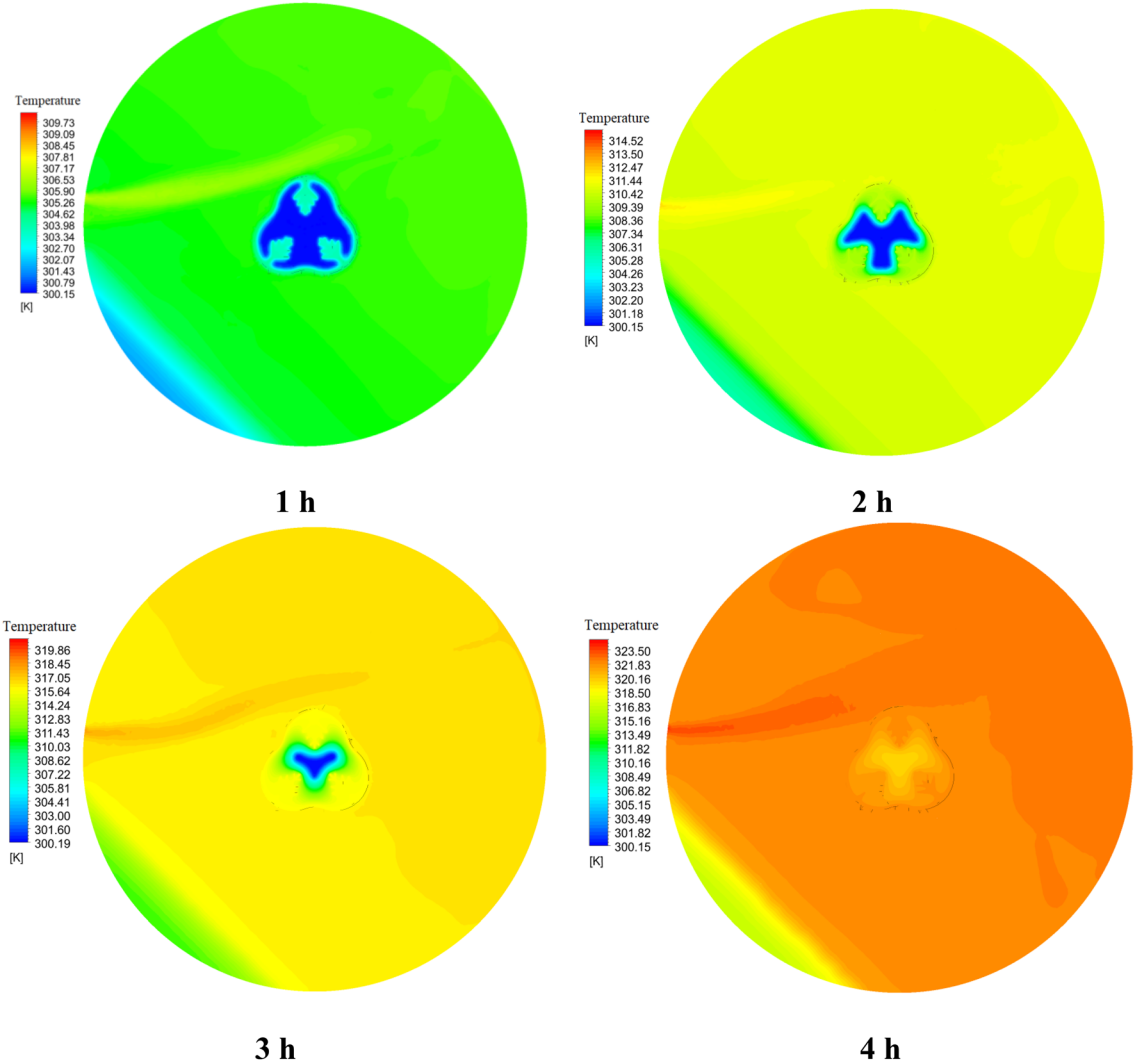
The temperature contour inside the tank at different times.

Figure 22 shows the temperature contour of the storage tank at different times for Case 8. The temperature contour demonstrates that the temperature is higher in the top part of the storage tank than in other parts. The fluid temperature is also the highest when it enters the storage tank. The PCM temperature contour in this Figure differs from the PCM temperature contour in Fig. 15 because this Figure uses the global mode in the CFD-POST software to display the temperature distribution contour for a nanofluid storage tank more clearly. However, the temperature legend in both figures is the same according to the contour colors. The temperature contour of the nanofluid storage tank shows that the temperature is lowest in the storage tank's lower left corner, which is the stagnant area, where natural convection flow does not happen.

### Energy storage analysis

**Figure 23:**
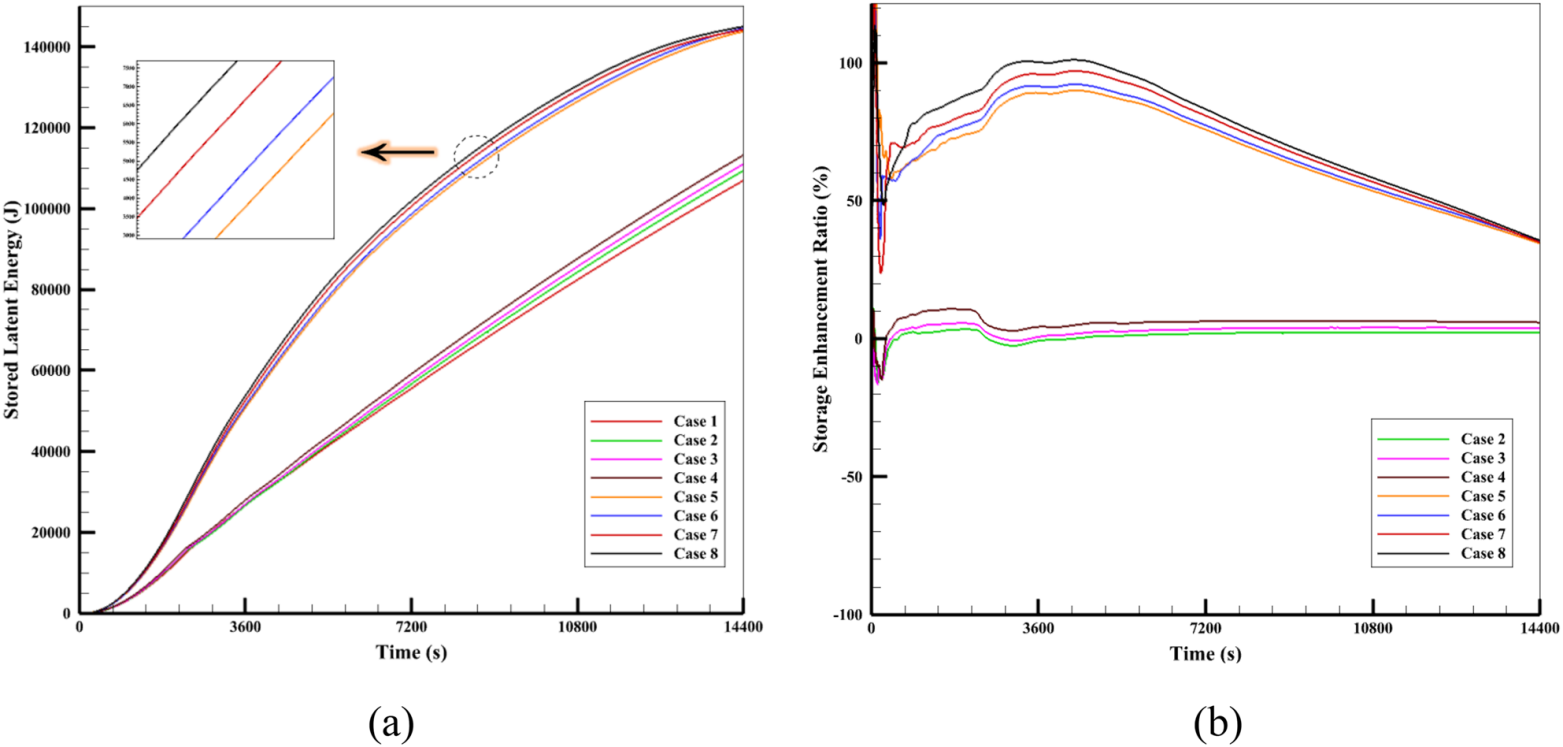
The comparison of different cases in terms of (**a**) stored latent energy and (**b**) storage enhancement ratio (SER) during a 4-h period.

This section examines the thermal energy stored in various simulated cases to obtain the maximum storage energy and the best system performance parameter. The combined ETCSWH system's latent heat storage capacity in the various modes that were discussed in the previous sections has been calculated. Figure 23a indicates the transient stored latent energy for various cases during the simulation period. The graph clearly shows that the highest amount of stored energy of the ETCSWH system for the case with NEPCM, nanofluid and fin (Case 8) is 145.047 kJ and the lowest amount of stored energy for the case without NEPCM, without fin and with water-base fluid is 106.928 kJ. The graph also shows that cases with fins store more energy than cases without fins. The graph designates that nanofluid, NEPCM, and the use of fins increase the stored heat in the ETCSWH system, respectively. The finned casing has a more noticeable impact on the heat response.

The use of fins, along with the addition of nanomaterial to PCM and water, leas to in a 35.7% increase in the latent energy stored compared to the simple case at the end of the fourth hour. It is possible that during the simulation, this percentage difference (Case 8 compared to Case 1) will be even more.

For example, in Case 8, which has the most energy storage, after one, two, three, and four hours, its stored energy is 100.3%, 83.3%, 58.4%, and 35.7% more than in Case 1, respectively. The use of fins contributes to the increased energy storage during the early hours, as it increases heat transfer to PCM, and more PCM melts and stores energy in less time. The Storage Enhancement Ratio (SER), as defined by Eq. 27^54^, is illustrated in Fig. 23b.

27 $$SE_{R} = \frac{{SE_{i} - SE_{1} (t)}}{{SE_{1} (t)}} \times 100\% ,\quad SE_{i} = \left\{ {\begin{array}{*{20}l} {\frac{{m_{pcm} C_{p,pcm} (T_{pcm,t} - T_{{pcm,t_{0} }} )}}{{t - t_{0} }},\quad t \le t_{1} } \hfill \\ {\frac{{m_{pcm} C_{p,pcm} (T_{pcm,t} - T_{{pcm,t_{0} }} )}}{{t_{1} - t_{0} }} + \frac{{\gamma m_{pcm} l}}{{t - t_{1} }},\quad t_{1} < t < t_{2} } \hfill \\ {\frac{{m_{pcm} C_{p,pcm} (T_{pcm,t} - T_{{pcm,t_{0} }} )}}{{t_{1} - t_{0} }} + \frac{{\gamma m_{pcm} l}}{{t_{2} - t_{1} }} + \frac{{m_{pcm} C_{p,pcm} (T_{pcm,t} - T_{pcm,melting} )}}{{t - t_{2} }},\quad t > t_{2} } \hfill \\ \end{array} } \right.$$

The above equation uses the subscript 1 for Case1 (reference Case) and the subscript *i* for the other cases (Cases 2–8). The mass and average temperature of PCM at time *t* are indicated by the letters $m_{pcm}$ and $T_{pcm,t} ,$ respectively. $\gamma$ also stands for liquid fraction. It measures the proportion of the reference case's stored latent energy to the difference in latent energy between the new case and the reference case at each time sample. Compared to the reference case, SER = 100% indicates a two-fold increase in heat storage, while SER = 0% means no improvement in energy storage. Figure 23b displays the SER for different Cases 2–8. According to the diagram, SER is almost equal to 100% between 3000 and 4850 s. For finned cases, a sudden initial rise in SER can be observed. This is due to the use of fins, which improve heat transfer and make more PCM melt in less time. However, as the storage process progresses, the enhancement ratio drops below 36% in the last hour. During the four hours of energy storage, Case 8 exhibits the highest SER. It reaches its maximum value of 101.14% in 4339 s. For Cases 2 through 8, the average SER time for 4 h of energy storage is 1.27%, 3.07%, 5.93%, 67.38%, 68.42%, 71.40%, and 74.22%, respectively. With a total of 4 h of storage, Case 8 performs the best among the cases.

## Conclusion

This research presents a numerical analysis of an ETCSWH system that uses a finned PCM chamber and nanofluid. The suggested system combines PCM and water-base fluid with 3% volume fractions of Al_2_O_3_ and Cu nanoparticles, respectively, to enhance heat transfer, accelerate the melting process, and enhance system performance. The analysis covers the PCM melting process, thermal energy storage, fluid flow behavior, speed distribution, and temperature contour in the storage tank and three sections of the absorber tube. The system is evaluated using CFD for a period of 4 h in a 3D transient simulation, with an angle of 45 degrees, which is the optimal angle for performance. The simulation results are validated and verified by numerical and experimental investigations, which show a strong correlation between the data. The significant outputs are:

- The results show that the cases with fins melt faster than the cases without fins. Case 8, which has nanofluid, NEPCM, and fins, enhances the melting process by 39.7% compared to Case 1, and Case 4, which has nanofluid and NEPCM, enhances it by 5.2% compared to Case 1, within 4 h.
- Case 8 is the best case that achieves complete melting in 12,681 s (equivalent to approximately 3.52 h) and also reaches complete melting 867 s (equivalent to approximately 15 min) earlier than Case 5, which is attributed to the presence of nanofluid and NEPCM. However, Case 1 only melts 71.58% of the PCM.
- Case 8 has the best performance and the highest rate of heat transfer, with a 43% and 6.4% reduction in PCM melting time compared to Cases 1 and 5, respectively. This is due to the use of nanofluid, NEPCM, and fins, which affect the melting process.
- Among the examined cases, Case 8 has the highest possible NEPCM temperature of 321.25 K compared to other cases, which results in more thermal energy storage.
- Case 8 with fins reaches the maximum liquid fraction of 0.7887 after 2 h of simulation, which is 70.46% higher than the case without fins (Case 4).
- The addition of Al_2_O_3_ nanoparticles to PCM reduces the melting time of Case 7 with finned NEPCM by 5%.
- By comparing different cases, the highest amount of stored energy of the ETCSWH system for Case 8 is 145.047 kJ and the lowest amount of stored energy is 106.928 kJ for the case without nanoparticles, without fins and with water-base fluid.
- The highest heat SER during 4 h of energy storage is related to Case 8, which reaches the maximum value of 101.14% in 4339 s.
- Case 8, which has the most energy storage, has 100.3%, 83.3%, 58.4%, and 35.7% more stored energy than Case 1, after one, two, three, and four hours, respectively. For Cases 2 through 8, the average SER time for 4 h of energy storage is 1.27%, 3.07%, 5.93%, 67.38%, 68.42%, 71.40%, and 74.22%, respectively.

## Data Availability

All data generated or analysed during this study are included in this published article.
